# New insights and advances on pyomelanin production: from microbial synthesis to applications

**DOI:** 10.1093/jimb/kuac013

**Published:** 2022-07-22

**Authors:** Faustine Lorquin, Philippe Piccerelle, Caroline Orneto, Maxime Robin, Jean Lorquin

**Affiliations:** Mediterranean Institute of Oceanology (MIO), Aix-Marseille Université, 163 avenue de Luminy, 13288 Marseille Cedex 9, France; Mediterranean Institute of Marine and Terrestrial Biodiversity and Ecology (IMBE), Aix-Marseille Université, 27 boulevard Jean Moulin, 13385 Marseille Cedex 5, France; Mediterranean Institute of Marine and Terrestrial Biodiversity and Ecology (IMBE), Aix-Marseille Université, 27 boulevard Jean Moulin, 13385 Marseille Cedex 5, France; Mediterranean Institute of Marine and Terrestrial Biodiversity and Ecology (IMBE), Aix-Marseille Université, 27 boulevard Jean Moulin, 13385 Marseille Cedex 5, France; Mediterranean Institute of Marine and Terrestrial Biodiversity and Ecology (IMBE), Aix-Marseille Université, 27 boulevard Jean Moulin, 13385 Marseille Cedex 5, France; Mediterranean Institute of Oceanology (MIO), Aix-Marseille Université, 163 avenue de Luminy, 13288 Marseille Cedex 9, France

**Keywords:** Pyomelanin, Polymerization, Hydroxylase, Laccases, Applications

## Abstract

Pyomelanin is a brown-black phenolic polymer and results from the oxidation of homogentisic acid (HGA) in the L-tyrosine pathway. As part of the research for natural and active ingredients issued from realistic bioprocesses, this work re-evaluates the HGA pigment and makes an updated inventory of its syntheses, microbial pathways, and properties, with tracks and recent advances for its large-scale production. The mechanism of the HGA polymerization is also well documented. In alkaptonuria, pyomelanin formation leads to connective tissue damage and arthritis, most probably due to the ROS issued from HGA oxidation. While UV radiation on human melanin may generate degradation products, pyomelanin is not photodegradable, is hyperthermostable, and has other properties better than L-Dopa melanin. This review aims to raise awareness about the potential of this pigment for various applications, not only for skin coloring and protection but also for other cells, materials, and as a promising (semi)conductor for bioelectronics and energy.

## Graphical Abstract

**Graphical Abstract:**
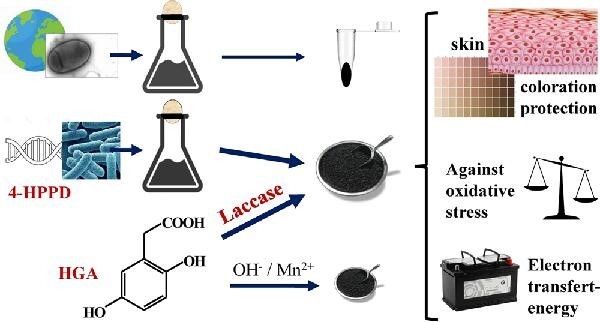

## Introduction

**Fig. 1:**
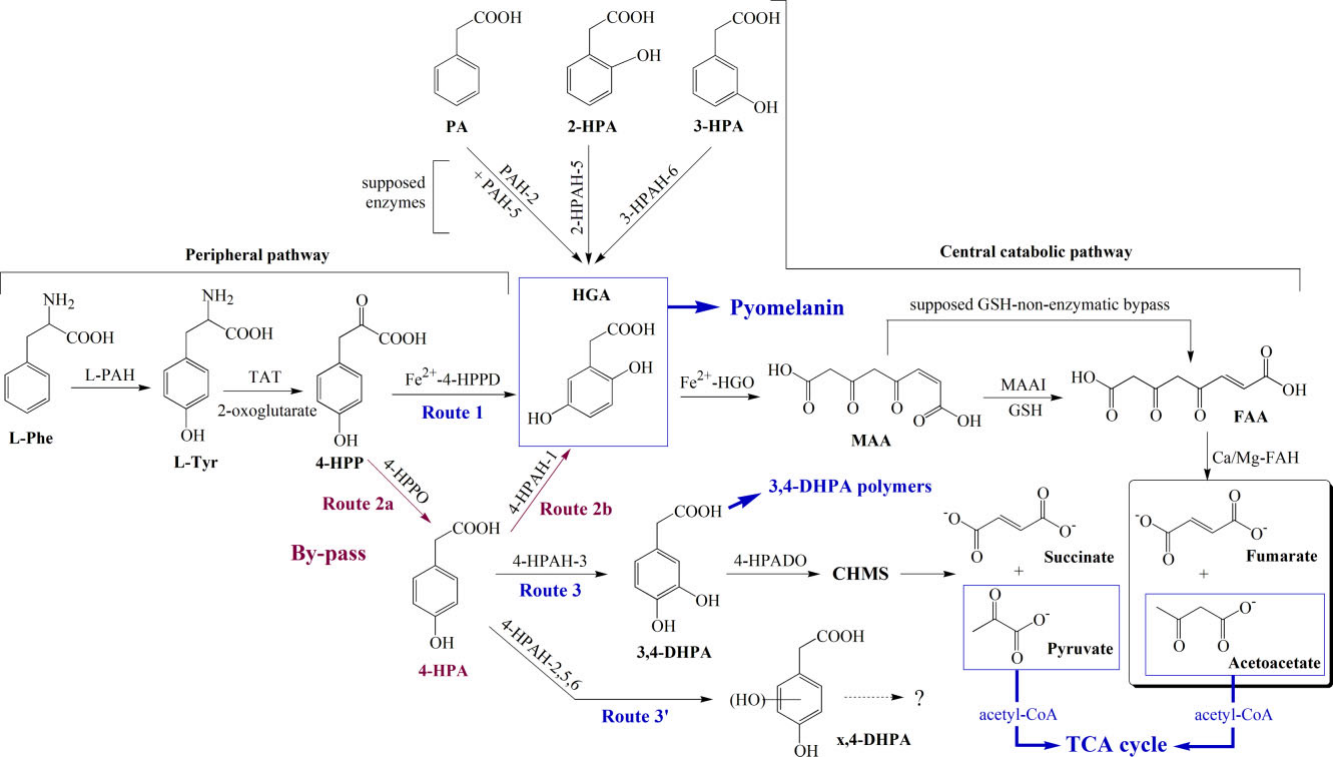
Biosynthesis of pyomelanin through the homogentisate pathways is redefined as the HGA catabolon. This catabolon is involved in the degradation of L-Phe, L-Tyr, PA, and two hydroxy-PA derivatives into the common HGA and further toward the same route of catabolic convergence until fumarate and acetoacetate. *Compounds*: CHMS, 5-carboxymethyl-2-hydroxymuconic semialdehyde acid; 3,4-DHPA, 3,4-dihydroxyphenylacetic acid; FAA, fumarylacetoacetate; FAH, FAA hydrolase; GSH, glutathione; HGA, homogentisic acid; HGO, homogentisic acid 1,2-dioxygenase; *x*-HPA, *x*-hydroxyphenylacetic acid (*x* = 2, 3, or 4); 3,4-HPADO, 3,4-dihydroxyphenylacetate dioxygenase; 4-HPAH-*y*, 4-hydroxyphenylacetate hydroxylase-(*y* = 1, 2, 3, 5, or 6); 4-HPP, 4-hydroxyphenylpyruvic acid; 4-HPPD, 4-hydroxyphenylpyruvate dioxygenase; 4-HPPO, 4-hydroxyphenylpyruvate oxidase; MAA, 4-maleylacetoacetate; MAAI, MAA isomerase; PA, phenylacetic acid; L-PAH, L-phenylalanine hydroxylase; PAH-*z*, phenylacetic acid hydroxylase-(*z* = 2 or 5); L-Phe, L-phenylalanine; TAT, tyrosine transaminase; and L-Tyr, L-tyrosine.

The true starting date is 1897 when a water-soluble, brown pigment-producing bacterium was isolated from a gummatous leg ulcer of a French cavalryman and was described as a “pyocyanic bacillus” by Maxime Radais at the Faculty of Pharmacy in Paris. In 1902, began the ever-rebounding story of alkaptonuria (ALK), a rare, devastating, and osteoarticular disease, which aroused a lot of passion in the Great Ormond Street Hospital in London, and later all over the world. Paternity of the pigment origin undoubtedly belongs to La Du et al. (1958) and Zannoni et al. (1969), who demonstrated the involvement of the enzyme and the oxidation of homogentisic acid (HGA, 2,5-dihydroxyphenylacetic acid) that led to the ochronotic pigment found in connective tissue from patients with ALK. Thereafter, Yabuuchi & Ohyama (1972) confirmed these results and proposed the term “pyomelanin” for the pigment produced by *Pseudomonas aeruginosa*. The mutation of the gene responsible was revealed only in 1994 (Janocha et al., 1994). Just after, studies on *Aspergillus nidulans* have shown that the Δ*hmg*A mutant bears some analogy to results with *A*spergillus *fumigatus*, and the transfer of results to humans confirmed that polymers in ALK are associated with the defect of the HmgA protein (Fernández-Cañon & Peñalva, 1995; Schmaler-Ripcke et al., 2009). Pyomelanin belongs to the heteroclite group of allomelanins encountered in all kingdoms in which melanins are produced from 1,8-DHN (1,8-dihydroxynaphtalene), 1,3,6,8-THN (1,3,6,8-tetrahydroxynaphtalene), catechols, GHB (γ-glutaminyl-4-hydroxybenzene; see Supplementary Material), and HGA (see Fig. 1). To date, the HGA polymer still raises many questions about its functions and structure. The pigment has retained a poor image since its involvement in ALK disease and the low amounts generally produced by microorganisms. For these reasons, neither a production process nor an application has been developed until very recently. So what is the most appropriate method to produce pyomelanin, and how to identify microbial candidates?

## HGA Pathways and Related Routes

Microorganisms are models of choice for HGA synthesis and production studies; some pathways have also been highlighted from studying mammalian cells with metabolic alterations to understand and potentially treat human pathologies. In the L-tyrosine (L-Tyr) degradation pathway, firstly L-phenylalanine (L-Phe) is converted to L-Tyr by the L-phenylalanine hydroxylase (L-PAH; EC 1.14.16.1), a non-heme iron-dependent enzyme. Then L-Tyr is converted to 4-hydroxyphenylpyruvate (4-HPP) by a tyrosine transaminase (TAT; EC 2.6.1.5) in the presence of 2-oxoglutarate as the cofactor. At this stage, two main distinct routes, 1 and 2 (Fig. 1), lead to HGA.

### The Two Main HGA Routes

In route 1, the most widely reported, 4-HPP is converted to HGA by a non-hemic but Fe^2+^-dependent enzyme, 4-hydroxyphenylpyruvate dioxygenase (4-HPPD, EC 1.13.11.27) (Raspail et al., 2011). Then HGA is transformed to 4-maleylacetoacetate (MAA) by a Fe^2+^-dependent 1,2-dioxygenase (or HGA oxidase, HGO, EC 1.13.11.5), the rate-limiting step of L-Tyr catabolism, which opens the aromatic ring by an *ortho*-cleavage mechanism (C_1_–C_2_ cleavage). MAA subsequently undergoes a *cis*-*trans* reversible isomerization catalyzed by a glutathione (GSH)-dependent maleylacetoacetate isomerase (MAAI, EC 5.2.1.2) and forming fumarylacetoacetate (FAA; Adachi et al., 1966). FAA is then hydrolyzed by a Ca/Mg-dependent FAA hydrolase (FAH, EC 3.7.1.2) in acetoacetate and fumarate, which will feed the Krebs cycle (Chapman & Dagley, 1962). In contrast, to human FAH, whose mutation causes hereditary tyrosinemia type I (TIT), MAAI failure does not produce disease. In addition, MAA can also be isomerized to FAA in the absence of MAAI, possibly by a GSH-dependent non-enzymatic bypass mechanism through succinylacetoacetate (Fernández-Cañon et al., 2002). From MAA, a GSH-independent isomerase has been described occasionally in a *Bacillus* sp. strain and was shown to form maleate (instead of fumarate) and acetoacetate (Crawford, 1976). In the case of a mutation (like in ALK) or inactivation of the HGO activity, the HGA rises in excess and is excreted from the cell, where it is oxidized rapidly in the presence of O_2_ to form pyomelanin.

In route 2 (2a and 2b), the least studied, 4-HPP is converted to 4-hydroxyphenylacetate (4-HPA) by a 4-HPP oxidase (4-HPPO, EC 1.2.3.13) (Blakley, 1977). At this stage, a NAD(P)H-dependent key enzyme, 4-HPA-1-hydroxylase (4-HPAH-1, EC 1.14.13.18) hydroxylates the ring at the C_1_ position. HGA is formed after transposition, named the ‘‘NIH shift’’ for the National Institute of Health, the research unit of origin; the shift concerns the acetic group -CH_2_-COOH, which moves to carbon C_2_ (Hareland et al., 1975; see the mechanism of hydroxylation in Supplementary Material). Then, the HGA ring is opened by the HGO enzyme leading to the synthesis of the end products, similarly to route 1. C_1_-hydroxylation and HGA degradation were demonstrated in *Delftia acidovorans* (formerly *Pseudomonas or Comamonas acidovorans*; Hareland et al., 1975), *Bacillus* sp. and *Moraxella* sp. (Crawford, 1976), *Vibrio cholera* (Kotob et al., 1995), *Shewanella colwelliana* (Coon et al., 1994), *Pseudomonas putida* (Arias-Barrau et al., 2004), *Xanthobacter* sp. (van den Tweel et al., 1986), *Flavobacterium* sp. (van den Tweel et al., 1988), *Azoarcus evansii* (Mohamed et al., 2002), *Cryptococcus neoformans* (Frases et al., 2007), *Halomonas olivaria* (Amouric et al., 2014; Liebgott et al., 2008), and many others. C_1_-hydroxylation is much less encountered than C_3_-hydroxylation, which is generally carried out by a 4-HPAH-3 enzyme in most microorganisms by forming 3,4-dihydroxyphenylacetate (3,4-DHPA; route 3, Fig. 1). This *ortho*-diphenol polymerizes much less rapidly than HGA in bacteria, such as *Klebsiella pneumoniae* (Gibello et al., 1995), *Serratia marcescens* (Trias et al., 1989), but not in *Halomonas* sp. HTB24 because of the concomitant presence of hydroxytyrosol (or 3,4-dihydroxyphenylethanol), another *ortho*-diphenol, and a powerful antioxidant (Liebgott et al., 2007, 2009).

### Other Routes and the Homogentisic Catabolon Concept

More rarely, hydroxylation on carbon other than C_1_ by other hydroxylases (HPAH, Fig. 1) had also been identified from HPA isomers other than 4-HPA. They involve more specific transport systems, usually generate minor dihydroxy compounds, and may reveal the multipotential capacity of some microorganisms in the degradation of xenobiotics. HGA was synthesized at low quantities from 2-HPA (C_5_ hydroxylation) in *Pseudomonas fluorescens* (Baggi et al., 1983) and *Rhodococcus erythropolis* (Suemori et al., 1996) and 3-HPA (C_6_) in *Trichosporon cutaneum* (Anderson & Dagley, 1980), *P. putida* U (Arias-Barrau et al., 2004, 2005), *R. erythropolis* (Suemori et al., 1996), *A. nidulans* (Ferrer-Sevillano & Fernández-Cañón, 2007), and *B*urkholderia *xenovorans* LB400 (Mendez et al., 2011). In addition, the random mutant of *Beauveria bassiana* fungus strain L6577 has been shown to synthesize HGA from phenylacetic acid (PA), 2-HPA, 3-HPA, and 3-methoxy-PA, but the yield of HGA has not been reported (Staudenmaier et al., 1999). Surprisingly, L6577 provides a conversion of 4-chloro-PA to 3 g L^−1^ of 4-chloro-HGA, a photographic developer and a very useful compound for the preparation of pharmaceuticals.

Aromatics can be degraded by a group of enzymes that belong to a complex functional catabolic unit, the catabolon that integrates different routes of the upper pathways. These routes generally catalyze the transformation of structurally related compounds into a common catabolite, in this case, HGA. Such a concept is novelty, defined here as the HGA catabolon encompasses all the routes involved in the transformation of L-Phe, L-Tyr, 4-HPP, PA, 2-HPA, 3-HPA, and 4-HPA into HGA, which is the common intermediate (Fig. 1). HGA is subsequently catabolized through a route of convergence, the HGA catabolon core, into general metabolites.

### Pathways Coexistence With Pyomelanin

At this date, routes 1 and 2 (Fig. 1) have never been reported to coexist in the same microorganism or species, most probably because the investigations have focused mainly on the 4-HPPD rather than the 4-HPAH-1 enzyme. The same observation has been made for routes 2 (HGA bypass) and 3 (3,4-DHPA formation). In contrast, routes 1 and 3 (through 2a) coexist in bacteria of the same species that possess an extensive advanced cell factory due to their metabolic plasticity and better adaptation to different environments. In the degradation of L-Tyr and biogenic aromatic amines, this is especially the case for *P. aeruginosa* (Cuskey & Olsen, 1988; Rodríguez-Rojas et al., 2009) and *P. putida* (Arcos et al., 2010; Paliwal et al., 2014). Secondly, in a few cases, the coexistence of several pathways and melanin types has also been encountered in the same organism or species. In bacteria and yeasts, two routes occurred, one tyrosinase-dependent involved in 3,4-dihydroxyphenylalanine (L-Dopa) synthesis and another tyrosinase-independent pathway, however, individually highlighted by the degradation of substrates added separately in the culture. As an illustrations, pyomelanin was formed from L-Tyr (Carreira et al., 2001; Kurian & Bhat, 2018), and L-Dopa melanin from L-Tyr (Ganesh Kumar et al., 2013) or L-Dopa (Apte et al., 2013), in *Pseudomonas stutzeri* and *Yarrowia lipolytica*. Besides that, the case of fungi remains particularly confusing. Starting from acetyl-CoA or malonyl-CoA, they generally synthesize melanin from 1,8-DHN by a laccase (Sapmak et al., 2015; Upadhyay et al., 2013). Alternatively, a few fungi like *C. neoformans* known to form melanin from exogenous substrates, produce pigmentation from L-Dopa, catechol, and other catecholamine precursors (epinephrine, norepinephrine) by laccase(s) (Garcia-Rivera et al., 2005; Williamson, 1994), as well as pyomelanin from HGA (Frases et al., 2007). And if L-Tyr is the precursor, it is first converted to L-Dopa and dopaquinone by tyrosinases, then to L-Dopa-melanin. As another case, from L-Tyr, the pathogenic fungi *A. fumigatus* produces DHN-melanin as a virulence factor and pyomelanin as a defense function (Heinekamp et al., 2013; Schmaler-Ripcke et al., 2009).

## Identify the HGA Pathway

### First Approach by the Relevant Metabolites

Identifying the routes through the degradative intermediates remains possible if growth is slowed down by depleting the C and N sources of the medium. The strain preferentially utilizes the aromatic compounds over glucose, their degradation can be followed along with the growth by RP-HPLC-DAD (reverse-phase-HPLC-diode array detector), RP-HPLC-MS (mass spectrometry coupling in electrospray ionization [ESI] mode), and/or GC-MS (gas–liquid chromatography-mass spectrometry in electron-impact ionization [EI] mode) of the derivatized metabolites (Liebgott et al., 2007–2009). Accurate identification is achieved by their respective retention time, UV–visible spectra, and *m/z* mass data, over all standards available and spectra from the databank (NIST or Wiley). Cultures must be induced by appropriate substrates assayed separately at concentrations not exceeding 1–5 mM in the medium: (i) L-Tyr or even tyrosol, a phenolic strongly accumulated in olive-wastewaters, for the global identification of the intermediates at different times, (ii) 4-HPP (route 1 or 2), (iii) 4-HPA (route 2b, 3, or 3’), and (iv) HPA isomers (HPA, 2-HPA, and 3-HPA) for other HGA routes and through the respective hydroxylases involvement (Liebgott et al., 2007–2009; Lorquin et al., 2021; Turick et al., 2009) (Fig. 1). HGA and pyomelanin were shown to increase in the presence of an excess of 4-HPA and 4-tyrosol during the growth of *Halomonas titanicae* and *Halomonas Olivaria*, respectively, these two bacteria being unable to grow in the presence of L-Tyr (Liebgott et al., 2008; Lorquin et al., 2021). Identical strategies had also been used in fungi and yeasts (Almeida-Paes et al., 2017; Schmaler-Ripcke et al., 2009). By HPLC-DAD analyses of the culture supernatant, 4-HPA (λ_max_ 274 nm), 4-HPP (λ_max_ 295_weak_, 311 nm), and HGA (λ_max_ 290 nm) must be searched first and confirmed by mass spectrometry (ESI or EI mode) to propose routes 1 and/or 2. Metabolites directly issued from the degradation of HGA might also be identified by ion-exchange HPLC-DAD, MAA, and FAA (λ_max_ 315–330 nm each, depending on the pH) (Bergeron et al., 2001; Chapman & Dagley, 1962). The increase of acetoacetate and fumarate amounts evaluated by either ion exchange HPLC-refractive index detection or GC-MS analyses will confirm the L-Tyr catabolism via HGA routes.

### By Inhibition of the Key Enzymes

**Fig. 2:**
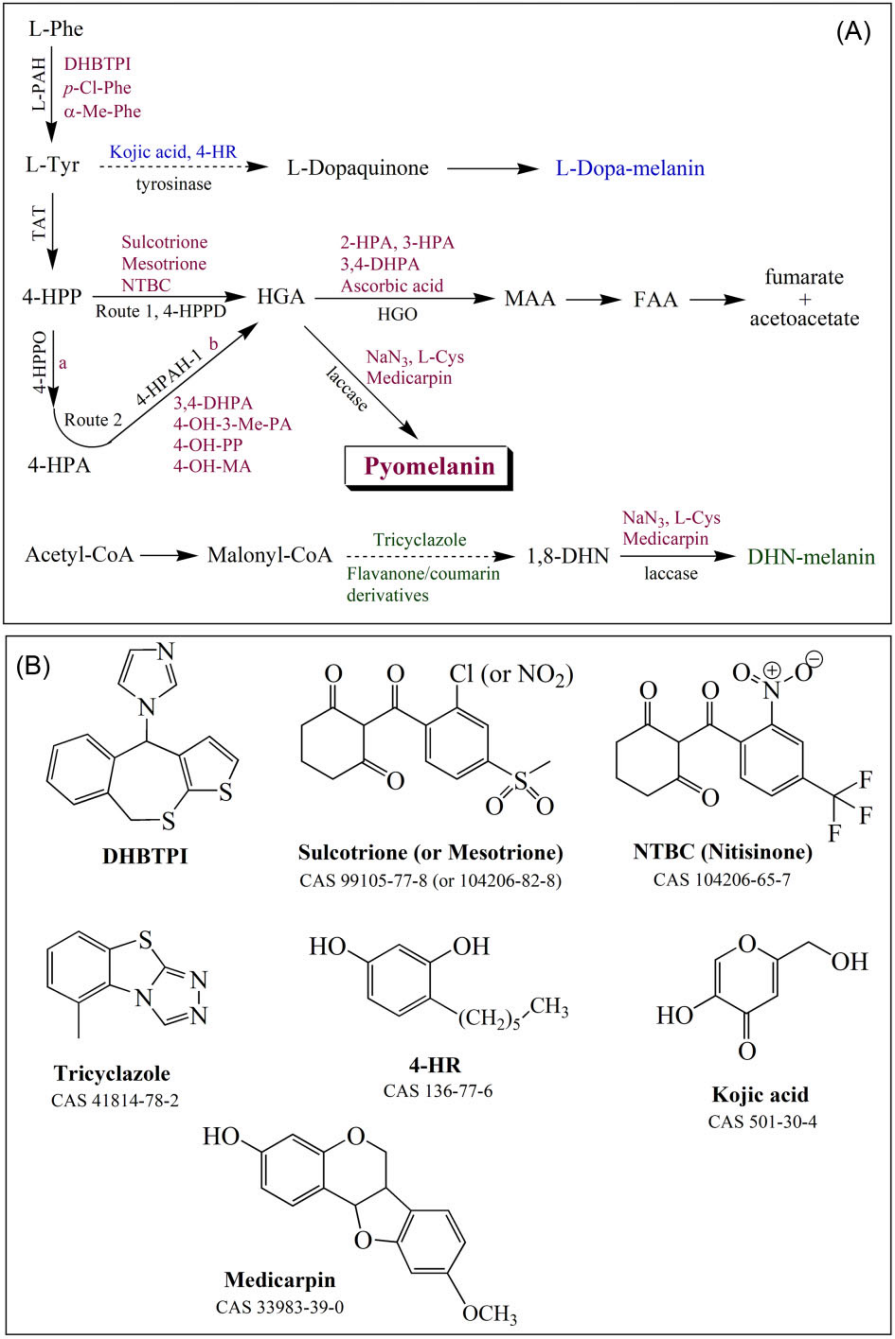
Inhibitors of pyomelanin synthesis and other inhibitors to differentiate pyomelanin from other melanin. (A) Inhibitors of pyomelanin synthesis are represented in red, compared to those of L-Dopa (blue) and DHN-melanin (green). Reactions are detailed in Fig. 1. (B) Chemical structure of the main inhibitors. *Abbreviations*: L-Cys, L-cysteine; DHBTPI, 1-(4,9-dihydrobenzo[e]thieno[2,3-b]thiepin-4-yl)-1H-imidazole; 4-HR, 4-hexylresorcinol; kojic acid, 5-hydroxy-2-(hydroxymethyl)-4H-pyran-4-one; MA, mandelic acid; medicarpin, 3-hydroxy-9-methoxypterocarpan; mesotrione, 2-[4-(methylsulfonyl)-2-nitrobenzoyl]cyclohexane-1,3-dione; NaN_3_, sodium azide; NTBC (nitisinone), 2-[2-nitro-4-(trifluoromethyl)benzoyl]cyclohexane-1,3-dione; PA, phenylacetic acid; PP, phenylpropionic acid; sulcotrione, 2-[4-(methylsulfonyl)-2-chlorobenzoyl]cyclohexane-1,3-dione; and tricyclazole, 5-methyl-1,2,4-triazolo(3,4-b)benzothiazole.

Inhibitors assayed separately are essential to decide between routes 1 or 2 (Fig. 1). As a global approach, better than *p*-chloro- and α-methylphenylalanine (Kelly & Johnson, 1978), a synthetic inhibitor acting as a small binder of the L-PAH enzyme, named DHBTPI (IC_50_ 3.8 μM; Aubi et al., 2015) must be assayed (see Fig. 2). DHBTPI does not affect the growth of *Legionella pneumophila* but abolishes the synthesis of pyomelanin. Experiments should be further organized around two ways: (A) inhibition of 4-HPPD (route 1) and 4-HPAH-1 (route 2b) enzymes; and (B) inhibition of HGO enzyme to promote HGA accumulation.

*Point A*. 4-HPPD (EC 1.13.11.27), the main described enzyme, is a Fe^2+^-dependent non-heme protein that belongs to the class of α-keto acid-dependent oxygenases with different roles in prokaryotes, plants, and animals. The enzyme has only two substrates, 4-HPP as the source of α-keto acid and molecular oxygen, and differs from most α-keto acid-dependent oxygenases that normally require α-ketoglutarate. The mechanism was suggested by Borowski et al. (2004), followed by works by Raspail et al. (2011) and Wójcik et al. (2014). In the last four decades and for weed control, agrochemical groups have identified new herbicides as potential 4-HPPD inhibitors that belong to pyrazoles, triketones, and isoxazoles (Ndikuryayo et al., 2017; van Almsick, 2009). Furthermore, these developments have made it possible to inhibit microbial 4-HPPDs successfully. Some inhibitors are derivatives of the natural phytotoxin leptospermone, and among them, nitisinone (or NTBC, see Fig. 2) was authorized in 2002 (USA) and 2005 (EU) to treat ALK, TIT, and hawkinsinuria diseases (Arnoux et al., 2015; McKiernan, 2006; Santucci et al., 2017). Finally, three inhibitors belonging to the triketone class, sulcotrione, mesotrione, and NTBC are commonly used to inhibit route 1 and rule on the involvement of 4-HPPD (Fig. 2). The 4-HPAH-1 enzyme (EC 1.14.13.18; route 2b) studies were initiated by those on gentisic acid (2,5-dihydroxybenzoic acid; Crawford, 1975; Crawford et al., 1975). The substrates are 4-HPA, NAD(P)H, and O_2_, and the activity needs FAD as the cofactor and Mg^2+^, the mechanism was never detailed afterward, but it was suggested by analogy to that of 4-HPAH-3 (Arias-Barrau et al., 2004; Liebgott et al., 2009; Prieto & Garcia, 1994). If Suemori et al. (1996) then Zink et al. (2012) purified the enzyme from *R. erythropolis* and *D. acidovorans*, respectively, ultimately, inhibition of 4-HPAH-1 was only reported by Hareland et al. (1975) and assumed mainly by 3,4-DHPA, 4-hydroxy-3-methyl-phenylacetic acid, 4-hydroxyphenylpropionic acid, and 4-hydroxymandelic acid (Fig. 2).

*Point B*. To accumulate pyomelanin, the inhibition or inactivation of the HGO activity would be a solution. The crystal structure of the human enzyme was first proposed by Titus et al. (2000) and the mechanism suggested by Borowski et al. (2005). Although HGO inhibition has not been studied extensively, the enzyme from various sources was successfully inhibited or inactivated by HPA analogs and could serve for other microbial HGO inhibition studies (Fig. 2 and Supplementary Material). Instead of using HPA analogs that might be incorporated and alter the structure of the final polymer, the absence of iron in microbial cultures is preferred to inactivate HGO (Liebgott et al., 2008).

*Doses range of the main inhibitors used.* Kojic acid (10–1000 μg mL^−1^), tricyclazole (10–100 μg mL^−1^), sulcotrione or mesotrione (50–100 μg mL^−1^; Fang et al., 2010; Tan et al., 2019), DHBTPI (10 μM; Aubi et al., 2015), 2- and 3-HPA (1–2 mM; Sugumaran & Vaidyanathan, 1978), 4-HR (10–100 μM; Dawley & Flurkey, 1993), medicarpin (0.1–0.5 mM; deduced from Martínez-Sotres et al., 2012, 2015), NaN_3_ (0.01–1 mM), L-Cys (0.1–1 mM; Couto & Toca, 2006), and NTBC (0.1–1 mM; Ketelboeter et al., 2014).

### Differentiate Pyomelanin From Other Pigments

In patients’ urine and as a suitable method for the diagnosis of ALK, the addition of NaOH plus hypochlorite accelerates the polymerization of HGA, showing two additional peaks at 406 and 430 nm in the visible spectrum and suggested to be HGA conjugated-bilirubin compounds (Tokuhara et al., 2018). In microorganisms, the possible coexistence of pathways and melanin might make the identification of pyomelanin among allomelanins and L-Dopa-melanin more complex. Inhibitors must help to differentiate the melanin types and pathways.

For an initial assessment, tricyclazole, an inhibitor of 1,8-DHN melanin synthesis (prevent the reduction of 1,3,8-THN and 1,3,6,8-THN), kojic acid, arbutin, and 4-hexylresorcinol (see Fig. 2), three tyrosinase inhibitors (L-Dopa pathway), are the most currently assayed in cultures, pyomelanin would not be affected by these treatments. Other flavanone and coumarin-derived compounds that inhibit DHN-melanin synthesis (Ganesh Kumar et al., 2011) and other tyrosinase inhibitors (reviewed in Chang, 2009) could be used as well. Secondly, the laccase inhibitors, Na-azide, L-cysteine, and the most recent medicarpin, may be assayed to prevent the HGA and/or DHN oxidation-polymerization, HGA and/or DHN would normally accumulate (Fig. 2). By such inhibition treatment, the excluded melanin must further be identified by FTIR (Fourier Transform InfraRed spectroscopy), ^13^C solid-state NMR (or ^13^C cross-polarization magic angle spinning NMR), alkaline-H_2_O_2_ (or permanganate) hydrolysis, and/or pyrolysis-GC-MS methods.

Some chemical features can also help to elucidate the melanin structures in a mixture. The very first difference is that eumelanin contains N, pheomelanin N and S, while allomelanins contain neither. Since pyomelanin and Dopa-melanin are dark-brown pigments, DHN-melanin is green-black, and GHB-melanin is reddish-brown. Moreover, the configuration of quinone or quinonimine residues is different from one class of melanin to another. It is *ortho* in L-Dopa-melanin, *para* in pyomelanin and GHB-polymers, and *meta* in 1,8-DHN melanins. Unfortunately, there are analytical brakes for the identification of pyomelanin, in contrast to L-Dopa melanin, for which compiled data of MALDI-ToF (matrix-assisted laser desorption/ionization-time of flight) mass spectrometry, NMR, and pyrolysis-GC-MS exist and complement degradative techniques (Pralea et al., 2019). Indeed, alkaline-H_2_O_2_ oxidation of L-Dopa melanin led to the identification of four possible degradation products, pyrrole-2,3-dicarboxylic acid, pyrrole-2,5,5-tricarboxylic acid, thiazole-4,5-dicarboxylic acid, and thiazole-2,3,5-tricarboxylic acid (d'Ischia et al., 2013; Wakamatsu et al., 2003). Such hydrolysis treatments failed for pyomelanin structure determination (Lorquin et al., 2021). A few researchers also tried to characterize pyomelanin by MALDI-ToF and ESI-MS-MS (tandem) mass spectrometry but gave inaccurate molecular weight and unknown fragments (Roberts et al., 2015; Singh et al., 2018). Consequently, priority must be given to the development of a specific matrix and ionization mode for the HGA polymer sequencing. Pyrolysis-GC-MS coupling methods also failed from pyomelanin of *Penicillium chrysogenum* (Vasanthakumar et al., 2015), and again from our HGA pigments (Lorquin et al., 2021), giving mainly 4-methoxybenzene acetic acid and 4-methoxybenzene propanoic acid, but no HGA. At least pyomelanin is negatively charged, resistant to acids, bleaching appears but not always when subjected to oxidizing agents, exhibits a positive reaction with FeCl_3_ and Na-dithionite, and is insoluble in most solvents. All that still rends the differentiation between melanin difficult. Its insolubility in organic solvents, however, may easier its purification from media that do not contain other melanin.

## *In Vitro* Polymerization and Transformations of HGA

**Fig. 3:**
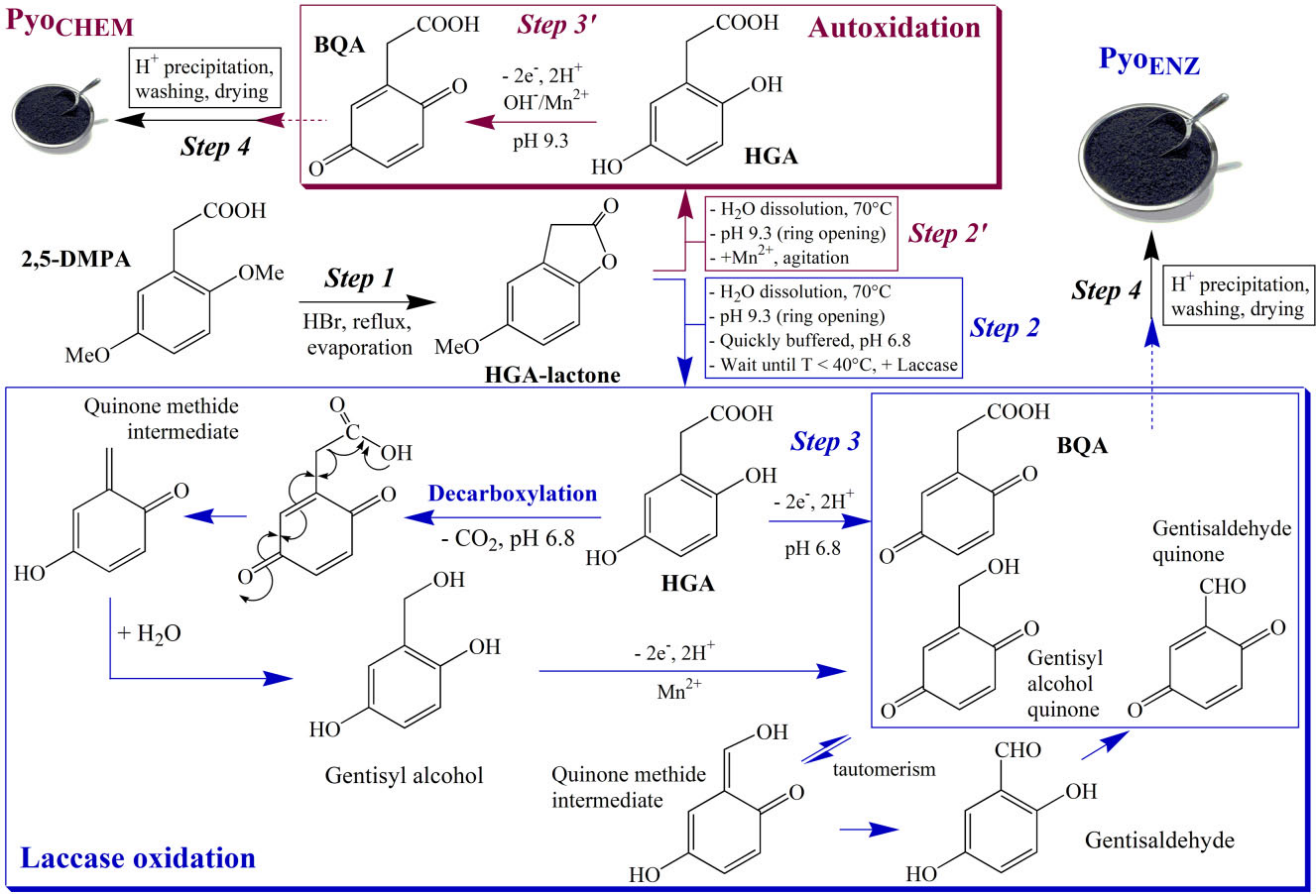
Schematic diagram of the production of pyomelanin by the laccase process (in blue) (reproduced with permission of Lorquin et al., 2021). Comparison to the abiotic autoxidation process (in red) via steps 2’ and 3’. The laccase oxidation generates a decarboxylation mechanism giving gentisyl alcohol and gentisaldehyde identified in the polymer. In step 1, 2,5-DMPA is demethylated by HBr at reflux and gives HGA-lactone at 99% yield. In step 2, the lactone is opened and the laccase is added at its optimal activity pH. In the case of the rMt laccase, the reaction medium is previously buffered at pH 6.8 (optimal activity). Step 4 is the final HCl precipitation, followed by washing and drying. *Compounds*: BQA, 1,4-benzoquinone acetic acid; gentisaldehyde, 2,5-dihydroxybenzaldehyde; gentisyl alcohol, 2,5-dihydroxybenzyl alcohol; 2,5-DMPA, 2,5-dimethoxyphenylacetic acid; and HGA, homogentisic acid.

For a long time, pyomelanin had been thought to be the result of the aerobic autoxidation of HGA from neutral to alkaline pH (Zannoni et al., 1969), followed by self-assembly into polymers. The oxidation is enhanced especially by Mn^2+^ that doubles the reaction yield, accelerated by SOD and Mn-pyrophosphate, and inhibited by NADH, GSH, and ascorbic acid (Coon et al., 1994; Martin & Batkoff, 1987). Formation of the initial oxidized product, 1,4-benzoquinone acetic acid (BQA), was proportional to the HGA concentration, optimal at 37°C, inferred by decreased UV-absorption at 290 nm (HGA) and increase at 250 nm (appearance of BQA). Toxic ROS, such as O_2_^.−^ HO**^.^**, and quinone radicals are rapidly formed and play a significant role in the etiology of ALK arthritis. While pyomelanin issued from the abiotic HGA autoxidation has been commonly reported (Ruzafa et al., 1995; Schmaler-Ripcke et al., 2009), the reaction had never been optimized until recently when an (HGA)/(Mn^2+^) ratio of 20 has been fixed for an optimal pyomelanin yield. By applying this ratio, an online process yielded 0.317 g of pyomelanin per g of 2,5-dimethoxyphenylacetic acid (2,5-DMPA, the starting compound; Table 1 and Fig. 3), a method that is easy to implement but limited to uses on a test lab scale. At alkaline pH, a chemical substitution at the C_4_ position of the BQA ring by added primary and secondary amines, had been described by Stoner & Blivaiss (1967) and might occur in biological systems where amino acids and proteins are present. By changing the pH from 7 to 4–5, BQA previously formed from HGA was shown to be chemically converted into 2,5-dihydroxybenzaldehyde (or gentisaldehyde) in the presence of Mn^2+^ or Cu^2+^ (Liebgott et al., 2008) (Fig. 3), whereas from HGA no reaction was observed at pH 6.5 (La Du & Zannoni, 1963). Gentisaldehyde is the resulting product of an oxidative decarboxylation reaction, a mechanism like that observed with 3,4-DHPA (Mefford et al., 1996).

**Table 1.** Summary of the Highest Pyomelanin Production Methods. *Value Recalculated from that Corresponding to bBioreactors Containing 7 L Leaches and 2 kg of Black Soldier Fly Larvae (See Text). 2,5-DMPA, 2,5-Dimethoxyphenylacetic Acid; YE, Yeast Extract; YPD, YE-Peptone-Dextrose; And YNB, Yeast Nitrogen Base

Microorganism or process	Expressed gene, mutagenesis type	Melanin yield	Phenolic precursor	C-N source	*T* °C	Reference
Pyomelanin						
HGA-Mn^2+^ autoxidation (optimized process)	–	0.317 g g^−1^	2,5-DMPA	–	30	Lorquin et al. (2021)
4-HPPD recombinant enzyme (from *Pseudomonas aeruginosa* PAO1 st.)	Heterologous expression, heat shock transformation	0.213 g L^−1^	L-Tyr	Glucose, casamino acids	37	Bolognese et al. (2019)
4-HPPD recombinant enzyme (from *Ralstonia pickettii*)	Heterologous expression in *E. coli* KSYH	0.315 g L^−1^	L-Tyr	LB medium with Cu^2+^	30	Seo & Choi (2020)
*Pseudomonas putida* F6 mutant (F6-HDO)	Random mutagenesis (transposon Tn5)	0.35 g L^−1^	L-Tyr	Na_3_-citrate, dextrose	30	Nicodinovic-Runic et al. (2009)
*Halomonas titanicae* (wild strain)	–	0.55 g L^−1^	4-HPA	Basal medium with YE	30	Lorquin et al. (2021)
*Yarrowia lipolytica* W29 (wild strain)	–	0.50 g L^−1^	L-Tyr	YNB medium with glucose, Asp, and Gly	30	Ben Tahar et al. (2019a)
Laccase extract polymerization (on-line optimized process)	Recombinant laccase from *M. thermophile* (rMt laccase)	1.25 g g^−1^	2,5-DMPA	–	30	Lorquin et al. (2021)
4-HPPD recombinant enzyme (from *Yarrowia lipolytica* W29)	Overexpressed gene (3 copies) by the JMY8032 strain construct	4.50 g L^−1^	L-Phe	YPD medium with glucose, Leu, uracil	37	Larroude et al. (2021b). See also construction in 2021a)
Pyomelanin-eumelanin (mixt)						
*Clostridium, Lactobacillus*, and flies (2 bioreactors process, see text)	–	5.35 g L^−1^*	–	Leachates, nutrient-rich solution	20	Popa & Nealson (2014)

## In Biological Systems: The Role of Laccases

Toxic ROS formed during the oxidation of HGA can deplete systemic or local antioxidants, leaving the remaining oxidants to increasingly react with essential proteins, DNA, and other macromolecules to cause or contribute to tissue toxicities (Hegedus & Nayak, 1994). In biological systems, like Mn^2+^, lactic acid, L-asparagine, and glycine promote coloration by contributing to the alkalinization of the medium and the autoxidation of HGA (Carreira et al., 2001). Another harmful effect concerns the cellular HGA oxidation at an alkaline pH in the presence of copper. Indeed, H_2_O_2_ can be chemically generated during Cu(II)-catalyzed HGA autoxidation and reacts with Cu(I) to form a Cu(I)-peroxide complex, this complex notably could cause oxidative DNA damage at thymine and cytosine residues (Hiraku et al., 1998). Is the chemical autoxidation of HGA the sole cause of its polymerization? While they exhibit similitudes with FTIR data (Schmaler-Ripcke et al., 2009) showing the best spectral resolution for lower size polymers, synthetic and microbial pyomelanin are structurally different (David et al., 1996; Lorquin et al., 2021; Zheng et al., 2013). Since polyphenol oxidases (PPOs) are copper enzymes, living cells also contain non-copper enzymatic systems able to catalyze phenol hydroxylation, such as cytochrome P450, 4-HPA, and 4-HPP hydroxylases, which may be considered as PPOs and which do not play a central role in physiological melanin formation. At pH 6.5, horseradish peroxidase could not polymerize HGA, while in the presence of GSH, the synthesis of 3,6-diglutathione-HGA occurred (La Du & Zannoni, 1963). Later, BQA had been shown to serve as a substrate for a partially purified copper-dependent oxidase from albino guinea pig skin (Zannoni et al., 1969). Logically suspected, tyrosinases were characterized in many melanin-producing microorganisms but have never been involved in HGA oxidation. Several authors hypothesized the action of laccases (EC 1.10.3.2) to polymerize *p*-dihydroxylated monophenols, as suggested in the bacteria *Vibrio cholerae* (Ruzafa et al., 1995) and *Alcaligenes eutrophus* (now *Cupriavidus necator*; David et al., 1996). In the wild-type *C. neoformans* B3501 strain, pyomelanin production was confirmed to be laccase-dependent by using a deleted construction (2E-TU Lac mutant), the enzyme expression is repressed by glucose that affects pigment induction (Frases et al., 2007). Pyomelanin-forming bacteria generally grow at pH 6–7, while the HGA autoxidation is optimal at pH 8–9, one more element in favor of the laccase(s) action. At least, a process working either at pH 6.8 or 5.0 with two recombinant laccases, one constructed from the *Myceliophtora thermophile* enzyme and expressed in *Aspergillus oryzae* (rMt), and the other from the *Pycnoporus cinnabarinus* enzyme, respectively, led to a similar and high production yield of pyomelanin (Lorquin et al., 2021). Most probably from BQA, enzymatic decarboxylation by the rMt laccase was highlighted and led to the partial formation of gentisyl alcohol (major) and gentisaldehyde (minor compound) identified by ^13^C solid-state NMR, which were integral parts of the polymer. These two decarboxylation-issued products represented 11–13% of the total components, in reverse order of level compared to the abiotic HGA decarboxylation described by Liebgott et al. (2008). Some remarks are needed here. The redox potential of HGA is 636 mV (Eslami et al., 2014), that of the rMt laccase 450–480 mV (Berka et al., 1997), but that of *P. cinnabarinus* laccase is 810 mV (Sigoillot et al., 2004), a value >636 mV, hence, a study that requires more investigations. The polymerization reaction by laccases does not require a mediator typically used for depolymerization; such a mediator also has no effect in the case of HGA polymerization by rMt laccase (Lorquin et al., 2021).

## Polymerization Mechanism and Structure Data

### Assembly Mode

**Fig. 4:**
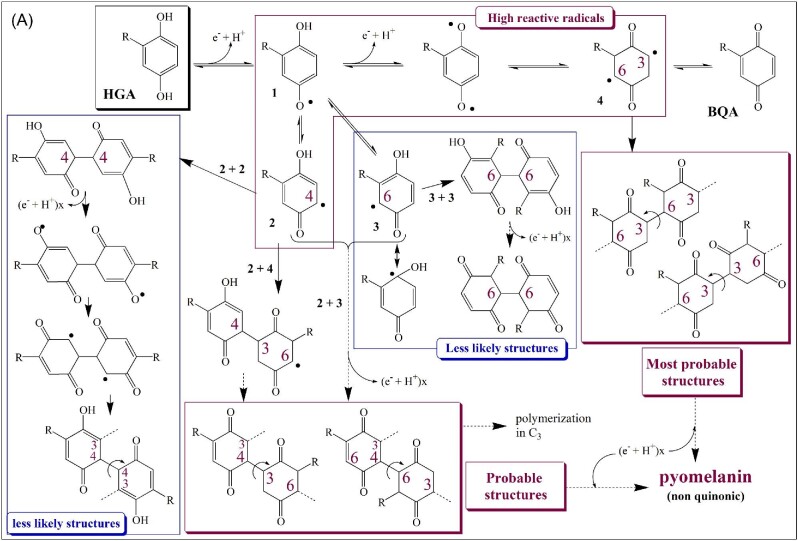

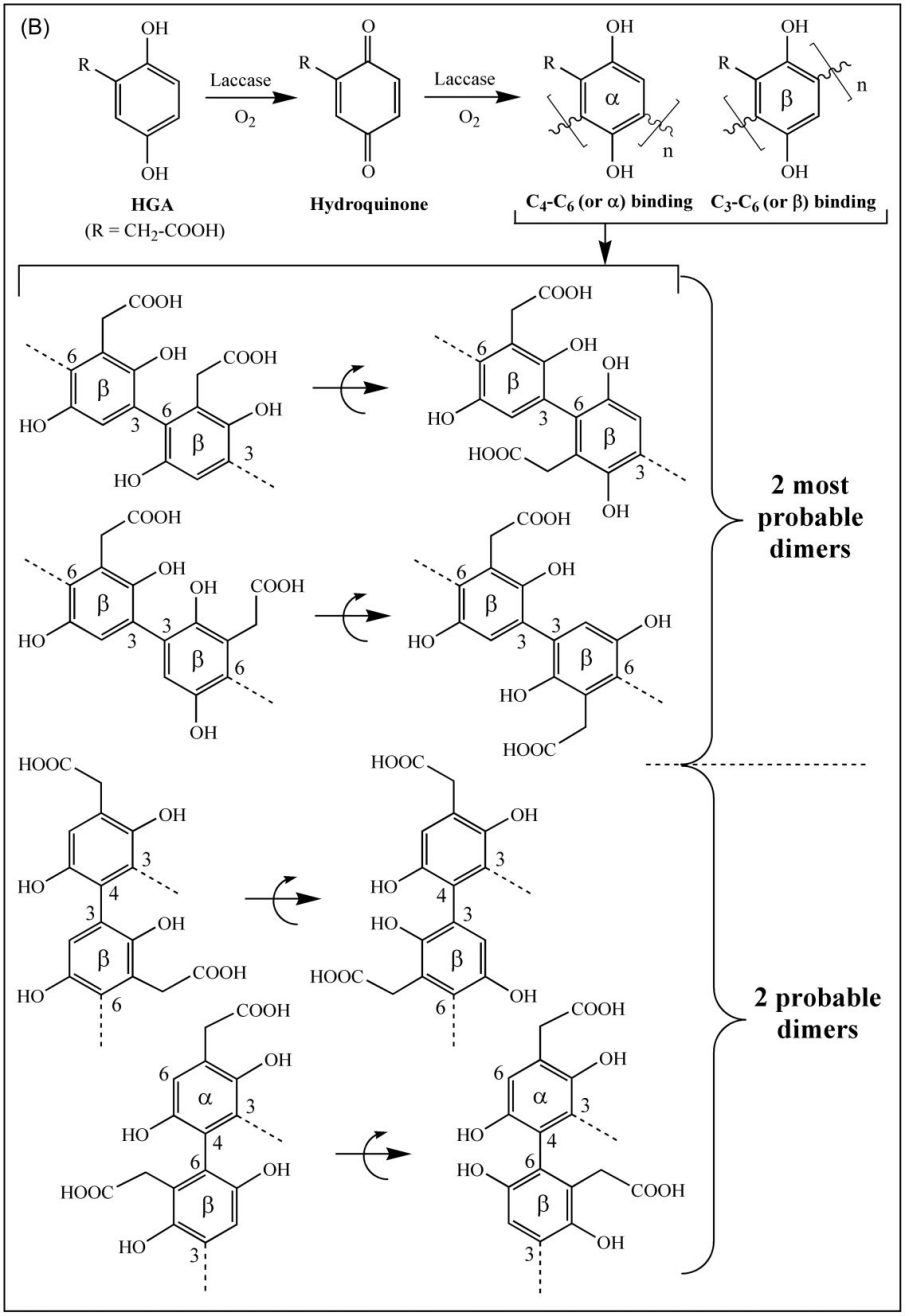
The proposed mechanism for the radical polymerization of HGA, showing the most probable structures (A, in red) and the detailed representations of the corresponding dimers in (B). *R* = -CH_2_-COOH. Gentisyl alcohol (major) and gentisaldehyde (minor) issued from the decarboxylation mechanism (laccase process, bacteria cultures) are not represented here but are incorporated into the polymer in the same manner as HGA radicals at locations of the chain that could not be determined at this time.

Despite the attempts of simulated 3D structures that were mapped with the Fukui function by Turick et al. (2010), FTIR, X-rays, mass spectrometry, and NMR techniques were never able to deliver consistent conclusions in the exact conformation and sequencing of pyomelanin, and self-aggregation studies were never completed. However, recent comparative studies have provided important insights. Whether for the synthetic (abiotic-alkaline), bacterial, or enzymatic (laccase) polymerization method, the high reactivity of the primary phenoxy radicals in favor of aryl radicals had been observed and confirmed the close analogy with the hydroquinone polymerization (Lorquin et al., 2021; Sun et al., 2013; Fig. 4A). The presence of C_ar_–C_ar_ linkages only and the total absence of C_ar_–O–C_ar_ (ether linkages) resonance in the ^13^C solid-state NMR spectra of the three polymers were also demonstrated. In the absence of suitable mass spectrometry and hydrolysis methods for exact monomer composition, the three structures were characterized by solid-state NMR and FTIR. The mechanism in Fig. 4A proposes the most and less probable dimer assemblies drawn from aryl radical generation and discriminated from their steric hindrances. Ultimately, from the C_4_–C_6_ (α-bindings) and C_3_–C_6_ (β-bindings) attachments on the HGA monomer, the eight resulting dimer configurations represented in Fig. 4B are the most advantageous arrangements less subject to steric effects and confirme the previous observations on hydroquinone polymerization (Sun et al., 2013). These eight structures let us imagine the multitude of possible combinations with these dimers forming the pyomelanin, which cannot be technically differentiated at this time. To summarize, the polymerization of HGA follows the same mechanism in the three types of procedure except that (i) gentisyl alcohol and gentisaldehyde are integral parts of the bacterial (Pyo_BACT_) and laccase (Pyo_ENZ_) polymers due to the decarboxylation by laccases (Lorquin et al., 2021), and (ii) the laccase yield is much higher than that of the chemical autoxidation. From the large dispersity value reported for the polymers, mainly Pyo_ENZ_ and Pyo_BACT_ (see next section), self-aggregation modes (π–π stacking, ionic bonds), and building blocks involved need to be elucidated.

## Molecular Weight and Spectroscopic Features

The size of pyomelanin was first evaluated from the pigment of the bacterium *A. eutrophus*, the molecular weight (*M*_W_) of 3000 Da estimated by GPC/SEC (gel permeation/size exclusion chromatography), however using unconventional polyethylene glycol as standard, while the synthetic pyomelanin (autoxidized HGA) was found at a smaller size of 1700 Da (David et al., 1996). In the absence of a suitable mass spectrometry technique, the GPC/SEC method on an adapted aqueous column remains the most appropriate, provided that an alkaline eluent ensures the solubility of the pigment to avoid high *M*_w_ agglomerates and the calibration line performed with similar polymers such as poly(styrene sulfonates) strongly recommended. Finally, the three optimized processes led to close *M*_w_ of 5400 (dispersity Ɖ 15.3) for the enzymatic (Pyo_ENZ_; general formula C_221_H_140_N_11_O_151_), 5700 Da (Ɖ 11.9) for the bacterial (Pyo_BACT_; C_193_H_179_N_15_O_187_), and a less high *M*_w_ of 2300 Da (Ɖ 6.64) for the autoxidized pyomelanin (Pyo_CHEM_; C_105_H_59_O_61_) (Lorquin et al., 2021). These sizes are higher than those of laccase-synthesized polymers of catechol (*M*_w_ 1268 Da), resorcinol (1489 Da), and hydroquinone (1157 Da) (Sun et al., 2013). Melanins have also paramagnetic characters, EPR (or ESR; electron paramagnetic [spin] resonance) techniques were successful to determine the free radical centers that absorb microwaves under a magnetic field, thereby generating a characteristic signal at 324–325 mT, for instance for pyomelanin of *C. neoformans* and *Rubrivivax benzoatilyticus* (Frases et al., 2007; Mekala et al., 2019). The UV–visible spectrum of pyomelanin is very wide (200–700 nm), the molar extinction coefficient is low, like that of eumelanin, from 7500 to 265 cm^−1^ mol^−1^ L for 200 to 820 nm, respectively (Sarna & Swartz, 2006). Sometimes, pyomelanin UV spectra could show an absorption in the 200–300 nm region that can be attributed to more complex structures and π → π* and *n* → π* transitions of amino, carboxylic, and aromatic moieties. Much less informative, other spectroscopic methods such as X-ray diffraction generate broad diffraction features due to the absence of regularly repeating units in polymers (Mekala et al., 2019).

## Properties of Pyomelanin

Despite the need to the extent its properties, pyomelanin has been distinguished from those of the largely available cuttlefish ink bag of *Sepia officinalis* (well-characterized by Magarelli et al., 2010) and synthetic Dopa-melanin (see preparation in d'Ischia et al., 2013), giving advantages to pyomelanin (Lorquin et al., 2021).

### Antimicrobial Activity

Pyomelanin is not essential for the growth of microorganisms but provides some advantages to their producers to cope with adverse challenges. *In vivo*, pyomelanin contributes to antimicrobial resistance and microbial pathogenesis as it is associated with virulence in a broad range of pathogenic fungi and bacteria (Nosanchuck & Casadevall, 2003). Notably, it was demonstrated that the HGO enzyme inactivation of *P. aeruginosa* and *B*urkholderia *cepacia* and the consequent pyomelanin accumulation led to a better adaptation to chronic infection in patients (Rodriguez-Rojas et al., 2009; Zughaier et al., 1999). The antibacterial and antifungal properties of the pigment purified from *P. aeruginosa* have been reported (Abdul-Hussien & Atia, 2017). However, the experiments were conducted on a few strains, which require more extensive investigations. The pyomelanin virulence also encompasses its action against antibiotics on which it binds, leading to the decrease of their antibacterial activities (Almeida-Paes et al., 2012, 2016; Liaw et al., 2010).

### UV-Antiradical Properties and (Photo)Stability

The main property of pyomelanin is to protect microorganisms from UV light limiting free radical (ROS) generation, increasing resistance to light, for instance in *Legionella* (Steinert et al., 1995). Generally, UVA induces damage by directly transferring energy or indirectly through ROS generated as primary and secondary radiolytic products (Maccarrone et al., 1997). The antiradical role of pyomelanin *in vivo* has been demonstrated in *P. aeruginosa* (Boles & Singh, 2008), *B. cepacia* (Keith et al., 2007), *A. fumigatus* (Schmaler-Ripcke et al., 2009), and many other strains. Pyomelanin has never been described as a producer of ROS under UV irradiation. The laccase-prepared pigment effectively scavenged ROS generated by UVA–visible irradiated keratinocyte with an IC_50_ of 82.2 μg mL^−1^, while the IC_50_ of synthetic melanin (Mel_SYNTH_, 284.1 μg mL^−1^) was higher than that of *S. officinalis* (Mel_SEPIA_) eumelanin very far (Lorquin et al., 2021). Comparatively, excitation of human eumelanin/pheomelanin by UV light produces ROS (singlet oxygen, superoxide, peroxide, and the hydroxyl radical) meanwhile they photoconsume O_2_ and are protective against skin cancer (Szewczyk et al., 2016). Human melanin transforms UV radiation (optical energy) into heat, a mechanism still not well understood with the sole link to the skin burning for lack of melanin. To this day, we do not know whether pyomelanin has this ability. If the human melanin particle size is one of the contributing factors to the variations in skin cancer rates among skin types (Nofsinger et al., 1999), size-dependent photoreactivity studies are needed for pyomelanin. Like human melanin, the HGA pigment displayed an electrochemical response to UV light when combined with a carbon paste electrode and reacted by offering protection from the free-radical formation (Turick et al., 2010). Human eumelanin and pheomelanin photochemically generate degradation products that are responsible for sunlight-induced melanoma formation by inducing cyclobutane-pyridine dimers from DNA (Premi et al., 2015). In contrast, strong UVA–visible irradiation at 550 W m^−2^ energy during 1 hr (200 J cm^−2^) on purified pyomelanin in solution, a dose much higher than the minima (72 J cm^−2^) imposed by the European Medicines Agency for photostability studies, did not generate degradation (Lorquin et al., 2021). Hence, pyomelanin is not a photosensitizing polymer, but is highly photostable and hyperthermostable (tested at 80°C for several days) due to the C_ar_–C_ar_ linkages constituting the polymer assembly.

### Against Oxidative Stress

The pigment diminishes the oxidizing stress of the host microorganism by its high tolerance to H_2_O_2_ and by a specific control due to several regulatory factors, as demonstrated in *Ralstonia solanacearum* (Ahmad et al., 2016). These findings reinforced previous results on oxidative stress from peroxide that decreased in clinical isolates of *P. aeruginosa* from infected patients because of pyomelanin synthesis (Rodríguez-Rojas et al., 2009).

### DPPH-Antioxidant Activity

The *in vitro* conventional DPPH (1,1‐diphenyl‐2‐picrylhydrazyl) assay was rarely reported due to its insolubility in organic solvents, and because the stable DPPH reagent reacts at slightly alkaline pH values. Despite this constraint, the purified pyomelanin from *Y. lipolytica* strain W29 at a low concentration in methanol was shown to exhibit a weak radical scavenging activity (EC_50_ 230 μg mL^−1^; Ben Tahar et al., 2019b). Further assays on the three types of HGA pigments prepared in DMSO along with two common L-Dopa melanins (Mel_SEPIA_, Mel_SYNTH_) were successful. They indicated that the laccase process-issued pyomelanin (Pyo_ENZ_, EC_50_ 27.5 μg mL^−1^) and the synthetic pigment (H_2_O_2_-oxidized L-Dopa, Mel_SYNTH_, EC_50_ 25.9 μg mL^−1^) have a DPPH-antioxidant activity equivalent to that of ascorbic acid (29 μg mL^−1^) (Lorquin et al., 2021), similarly to pyomelanin from *P. stutzeri* strain BTCZ10 and *Pseudoalteromonas lipolytica* BTCZ28 cultures (Kurian & Bhat, 2018; Narayanan et al., 2019). Barely better, the EC_50_ of pyomelanin optimally obtained from autoxidized HGA (Pyo_CHEM_) was 20.0 μg mL^−1^. These EC5_0_ values are far from that of pyomelanin of *H. titanicae* (130.0 μg mL^−1^) and the abundant eumelanin of *S. officinalis* (Mel_SEPIA_, IC_50_ > 300 μg mL^−1^) (Lorquin et al., 2021).

### Electron Transfer and Fe^3+^-Reducing Activities

Due to its redox cycling nature, *in vivo* as well as *in vitro*, pyomelanin was demonstrated to serve as a terminal electron acceptor, electron shuttle, or conduit for electrons (Turick et al., 2008a, 2010; see applications below). Consequently, pyomelanin increases the current response of biofilms for electricity production in microbial fuel cells. Moreover, microbial melanin synthesis might be a mechanism to mobilize cations from the ecosystem and store them. All melanins in the reduced form can anaerobically reduce ferric to ferrous oxide. Pyomelanin was shown to be able to transfer electrons, in the reduction of soluble FeIII in FeII, and especially to ensure homeostasis and an appropriate Fe^2+^/Fe^3+^ ratio for their survival, particularly when transporters and siderophores are absent (Andrews et al., 2003; Turick et al., 2002, 2003). In *L. pneumophila*, both HGA and pyomelanin mediate Fe^3+^ reduction, the resulting Fe^2+^ being available to the bacterium for uptake (Zheng et al., 2013). Under low dissolved oxygen levels, the HGA pigment accelerates the solid-phase metal reduction and aids in the survival of *Shewanella oneidensis* MR-1 (Turick et al., 2009). By an adapted ferrozine assay, the laccase-processed polymer (Pyo_ENZ_), HGA-autoxidized pigment (Pyo_CHEM_), and synthetic L-Dopa melanin (Mel_SYNTH_) exhibited a similar and highest Fe^3+^-reducing activity evaluated for Pyo_ENZ_ at 5.30 ng Fe^2+^ hr^−1^ μg^−1^ pyomelanin (Lorquin et al., 2021).

### A Moderate Anti-Inflammatory Activity

Generally, minimizing ROS minimizes inflammation in mammalian cells. By the LPS-activated murine macrophage RAW 264.7 cells to promote NO^.^ radicals as the anti-inflammatory assays, the only recorded result is that of the pyomelanin isolated from the *P. lipolytica* BTCZ28 strain and conditioned under ultra-small pyomelanin nanogranules (PNG; Narayanan et al., 2019). The pigment showed a moderate NO^.^ reduction of about 30% for 100 μg mL^−1^ of PNG, and in a complementary way, inhibition of cyclooxygenase (IC_50_ 95.5 μg mL^−1^), lipoxygenase (IC_50_ 88.9 μg mL^−1^), and myeloperoxidase (fivefold diminution of the activity at 100 μg mL^−1^) were also reported from the cell lysate of this strain, giving a proof-of-principle of using pyomelanin to diminish inflammation in therapeutic applications.

## Pyomelanin Production

Previous sections have highlighted that the production of pyomelanin by abiotic HGA autoxidation could not exceed 0.317 g g^−1^ 2,5-DMPA (Table 1), and as formerly suggested, seems unable to compete with microbial culture processes (David et al., 1996; Ruzafa et al., 1995). That might be explained in part by ^13^C solid-state NMR experiments that revealed a loss (∼40%) of carboxylic moiety without observable by-product degradation during the alkaline autoxidation of HGA-Mn^2+^ (Lorquin et al., 2021). With the absence of an efficient coordination catalyst able to prevent this loss and the feasibility of microbial and enzyme-catalyzed pyomelanin syntheses, several strategies may be proposed.

### Processes Using Wild Type Cells

Despite the great number of published pyomelanin-producing microorganisms, few works quantified HGA-melanin. Faced with the small quantities furnished, Turick et al. (2010) estimated they may produce between 1 and 115 femtograms of pyomelanin per cell, a 1–10 fg yield seems more common, which would approximately correspond to 0.03–0.3% pyomelanin relative to dry weight. A microbial development might logically use L-Tyr or 4-HPA supplemented wild-cultures, the absence of iron may prevent HGO and 4-HPPD activities while promoting that of the 4-HPAH-1 when present, and vice versa. Furthermore, instead of using organic HGO inhibitors that might polymerize with pyomelanin and affect its properties, other ions than iron should be selected to inhibit the HGO enzyme (see Table in Supplementary Material). Precautions must also be taken when using ascorbic acid or other reducing agents that activate or inhibit the HGO activity (Adachi et al., 1966; Schmidt et al., 1995; Veldhuizen et al., 2005). A pH-dependent reduction or oxidation of the iron atom bound to the enzyme site has been suggested but not yet well controlled. Among the genus *Halomonas*, HGA producers are easily cultivable strains at low NaCl concentrations in the presence of reduced N and C sources, and overproduce pyomelanin from 4-HPA by preculture inductions in the absence of Fe^2+^ (Liebgott et al., 2008). An induced and optimized culture of *H. titanicae*, a γ-proteobacterium isolated on the Titanic wreck, was also shown to synthesize the pigment through the 4-HPAH-1 bypass, however with a great amount of 4-HPA, at a yield equivalent to that of the yeast *Y. lipolytica* cultivated with L-Tyr (Table 1). At least an original patented process developed by Popa & Nealson (2014) furnishes a mixture of pyomelanin and eumelanin (% composition not given) from two controlled primary and secondary reactors in series, by repetitive trophic cycling with fermentation leachates or other low-cost nutrient-rich solutions. Nutrients are cycled between bacteria of the genera *Clostridium* and *Lactobacillus*, and black soldier fly larvae, *Hermetia illucens*, where polysaccharides are transformed into natural melanins (detailed in Supplementary Material). The patent reports that bioreactors containing 7 L of primary leachate, 2 kg of larvae, and incubated for 12–14 days at 20°C, produce a 30–37.5 g melanin mixture (Table 1).

Comparatively, the two main melanins are produced in much larger quantities. From L-Tyr, L-Dopa melanin yielded 27.9 g L^−1^ by the basidiomycete *Armillaria cepistipes* (Ribera et al., 2019). The production of DHN-melanin from glucose reached the highest melanin yield at 50 g L^−1^ by the *Vibrio alginolyticus* MMRF534 strain (Vijayan et al., 2017).

### Engineered Cells

The random mutagenesis (transposon Tn5) on the *P. putida* strain F6 generated mutants from which one has the gene encoding HGO disrupted. However, random mutagenesis on unknown sites is limited by the rational strategies used for further strain improvement as well as the phenotypic instability. As an important tool but never exploited for production, mutagenesis of the HGO enzyme (Δ*hmg*A) in iron-supplemented media has been demonstrated in *A. nidulans* (Fernández-Cañon & Peñalva, 1995), *A. fumigatus* (Schmaler-Ripcke et al., 2009), and *Bacillus thuringiensis* (Tan et al., 2019). Overexpression of the 4-HPPD gene in an *E. coli* strain cultivated on a simple C source (glucose, glycerol, and L-Tyr) remains the appropriate strategy (reviewed in Martínez et al., 2019). *E. coli* possesses endogenous aromatic amino acid transaminase, TyrB enzyme, and is HGO (-). A cosmid library had been generated from the marine bacterium *S. colwelliana* known to produce L-Dopa in addition to HGA, into *E. coli* from which one transformant produced melanin, the DNA sequences allowed the identification of the 4-HPPD (Fuqua et al., 1991). Many recombinant 4-HPPDs have further been developed in *E. coli*, such as those from *P. fluorescens* (Borowski et al., 2004; Wójcik et al., 2014), plants such as *Arabidopsis thaliana* and carrot (Lederer & Böger, 2005; Raspail et al., 2011), and human cDNA constructs to find effective inhibitors against ALK (Aarenstrup et al., 2002; Neuckermans et al., 2019). From these publications, no pyomelanin yield had been reported until the recent heterologous expression of the 4-HPPD gene from *Ralstonia pickettii* (Seo & Choi, 2020; Table 1). Much more effective, the gene 4-HPPD of the yeast *Y. lipolytica* was overexpressed in the chassis strain with up to three copies, leading to the highest yield of pyomelanin (4.5 g L^−1^; Table 1) and revealing that the yeast could convert L-Phe to L-Tyr or that the HGA pathway is strongly induced by L-Phe (Larroude et al., 2021b). Comparatively and from L-Tyr, note that an engineered *mel*C (tyrosinase gene) transformed in *Streptomyces kathirae* produced L-Dopa melanin at a yield of 28.8 g L^−1^ (Guo et al., 2015).

Let us now consider the bypassing route 2b through the 4-HPAH-1 enzyme (Fig. 1). This route exists in microorganisms, while the 4-HPPD path appears unique in plants, where 4-HPA is rarely identified, it might be a growth regulator. So far, no 4-HPAH-1 mutant has been developed. Gene sequence and cloning of the *D. acidovorans* enzyme had been given for the first time by Zink et al. in 2008 (Bayer's group patent, updated in 2012). The protein showed two components. The first one is *hpa*H, which codes for a flavoprotein NADH oxidase-dependent that transforms 4-HPA into a non-identified metabolite called Z; and the second is *hpa*C (HPAC), a peptide also designed as a 1,4-dihydroxyphenylacetate mutase (EC 5.4.99.-; Raspail et al., 2011) catalyzes the conversion of Z into HGA. To make plants tolerant to herbicide treatments, the strategy of Bayer's group was to implement the pathway of metabolic bypassing of 4-HPPD, by cloning in the plant the 4-HPAH-1 enzyme through *hpa*H and *hpa*C from *D. acidovorans* (Hareland et al., 1975) together with the 4-HPPO from *Arthrobacter globiformis* (Blakley, 1977). However, no HGA formation has been found from these constructs. In our laboratory, *hpa*H and *hpa*C genes from *Ruegeria pomeroyi* DDS3 and *H. olivaria* strains were cloned in *E. coli* BL21 (unpublished). Unfortunately, no HGA had been detected from the transformant cultures containing 4-HPA, suggesting that another DNA sequence might be involved in restoring the hydroxylase activity. Cloning an active 4-HPAH-1 would be further investigated regarding the low-cost 4-HPA substrate that would directly be converted to HGA with less energy consumption.

### Process Using Laccase

Laccases are responsible for the oxidation-polymerization of HGA (see previous sections). Hence, a simpler enzymatic process can replace microbial cultures, avoiding the constraints of sterility and control, allowing higher temperatures (laccases may work at 50°C), and is the ideal method for products that require predictable quality and redox properties. The current price of HGA proposed by suppliers in the world has become overestimated, about €800 per gram. Generally, the chemical syntheses of HGA are not applicable for a large-scale process because of the high cost of the starting reagents, the number of steps, and the final yield between 50 and 70% when indicated (Bloomer & Damodaran, 1980; Bostock & Renfrew, 1978; Leaf & Neuberger, 1948; Prasad & Adapa, 1992). Finally, the former procedure of DeForrest Abbott & Doyle-Smyth (1949), starting from 2,5-DMPA and through HGA-lactone, remains the most convenient, in a three-step online process, which can be widely large-scale extrapolated (Fig. 3). When using the concentrated extract of the rMt laccase, a high pyomelanin yield was obtained (Pyo_ENZ_; Table 1). The 2,5-DMPA could as well be easily prepared from 2,5-dimethoxyacetophenone, four times less expensive, by a Willgerodt–Kindler reaction.

## Applications: Toward Realistic Uses

### General Considerations

The degradation of pyomelanin by enzymes or microorganisms has never been reported to date. The main brake would be the pharmacological administration of the pigment further in contact with connective tissues. In this respect, it has been suggested that mono and small oligomers of BQA associated with oxidative stress would define the “ochronotic pigment” in ALK (Arnoux et al., 2015). What would happen if higher-sized pyomelanin such as Pyo_ENZ_ is administered, while in this case, there is no generation of free radicals and no arthritis by analogy to ALK. Waiting for investigations on the impact of high-size polymers on connective tissues, health applications are thus limited to cosmetology. On the other hand, is the pigment solubility an issue? Pyomelanin is not a soluble pigment; very fine particles are generally in suspension in neutral aqueous and most organic media. While waiting to find a pharmaceutic safety solvent, the only alternative remains to dissolve the dried pigment at 10 mg mL^−1^ in 0.05 N NaOH, or in special cases at 0.5 mg mL^−1^ in DMSO, which are the max. dissolved concentrations for preparing stock solutions (Lorquin et al., 2021). Moreover, the addition or presence of polyethylene glycol, proteins, lipids, and amines or amino acids just before the HGA polymerization might improve pigment solubility (Aravindakshan & Haberman, 1998; Stoner & Blivaiss, 1967). Note that melanin derived from natural sources, and most likely pyomelanin, generally shows chemical versatility by conjugation with other molecules, however the advantage of good biocompatibility with no antigenic response in living cells (Araújo et al., 2014; Bettinger et al., 2009).

### Applications in Cosmetology: Toxicological Aspects and Perspectives

Cytotoxicity is generally evaluated through two standard and complementary procedures (cell viability and acridine orange/ethidium bromide coloration) toward two mammalian cells, namely, mouse fibroblasts (NIH3T3) and human keratinocytes (HaCaT), that are used as a part of the 3D skin model for the assessment of the toxic hazard of cosmetic ingredients (Pfuhler et al., 2014). By these methods, pyomelanin extracted from the yeast *Y. lipolytica* was found as non-cytotoxic at 100 μg mL^−1^ (Ben Tahar et al., 2019b), and the same pigment-based gold nanoparticles showed neither cytotoxicity effect nor altered cell morphology (Ben Tahar et al., 2019a). Narayanan et al. (2019) also showed that pyomelanin from *P. lipolytica* strain BTCZ28 was a non-toxic pigment, which exhibits only ∼25% cell death at 100 μg mL^−1^, on fibroblast L929 cell line and by using the MTT (3-(4,5-dimethylthiazol-2-yl)-2,5-diphenyltetrazolium bromide) assay. Cytotoxicity toward human keratinocytes of Pyo_ENZ_ (laccase), Pyo_BACT_ (bacterial), and Pyo_CHEM_ (autoxidized HGA process), evaluated by the vital dye neutral red penetration technique, was found non-cytotoxic until 500 μg mL^−1^ (Lorquin et al., 2021). Studies have also been conducted for the action of pyomelanin on cancer cells *in vitro*. Purified pyomelanin from cultures of *P. aeruginosa* strains isolated from patients was assessed on lung A549 (IC_50_ 410.1 μg mL^−1^), skin A375 (234.4 μg mL^−1^), macrophage RAW264.7 (235.4 μg mL^−1^) cancer cell lines, and Vero (kidney, 100 μg mL^−1^) cell line, and showed moderate cytotoxicity with a dose-dependent effect (Mahmood et al., 2016). Much better, the tumor cell kill-effect of pyomelanin from the strain *P. lipolytica* BTCZ28 was estimated in the lung carcinoma cell line A549 with an IC_50_ value at 96.1 μg mL^−1^ (Narayanan et al., 2019). In summary, pyomelanin appeared non-cytotoxic against fibroblast and keratinocyte, and cytotoxic against cancer cell lines two characteristics of importance for skin health.

While two cases of melanin are used in cosmetics, a catechol-melanin from black olive fruits against sunburns and aging (Laboratoires Biocyte, France), and a non-communicated bio-melanin in sunscreen lotions (Baar Products, PA, USA), to date, pyomelanin has never been used in emulsion or other formulation types. Yet the pigment had been proposed for a long time as a potential ingredient in cosmetics and nanocosmetics for which we must address the aesthetics, conservation of the formulation, and its actual effect on the skin. While the brown color might seem unattractive, in emulsions the diluted pigment exerts a wood color and a natural aspect. It might also impart a natural tanned appearance when applied concentrated. The anti-inflammatory property of pyomelanin against skin disorders, even if moderate (see properties), is an excellent pharmacological complement. The pigment might treat post-inflammatory hypopigmentation as well, for instance in the cases of eczema, acne, trauma, burns, and psoriasis, as already suggested for mixtures of synthetic L-Dopa-melanin (Pawelek et al., 1995). In addition, due to its poor solubility notably, at pH 6, which is the pH of the skin and emulsions in general, there is a very high probability that high size pyomelanin does not permeate the stratum corneum barrier and reach the epidermis and dermis. Pyomelanin could also be formulated as an antimicrobial agent, a 0.05–0.1% max. concentration seems the most appropriate, as assayed in a sunscreen preparation containing a bacterial pigment (Kurian & Bhat, 2018). Extensive antibacterial assays related to skin infections, such as acne and nosocomial bacteria, have become necessary.

Considering the action of pyomelanin against light radiation and contrary to what one might think, pyomelanin as well as L-Dopa melanin in no way is sunscreen because of the low sun protection factor (SPF) from 2 to 4 (Epstein et al., 2007). SPF must be especially evaluated with a spectrophotometer equipped with an integrating sphere accessory to collect the scattered light from poly(methyl-methacrylate) plates. However, pyomelanin was shown to slightly enhance the SPF value (Ben Tahar et al., 2019b; Kurian & Bhat, 2018). The pigment absorbs UV radiation notably UVA without being degraded, even by heat, and effectively scavenges ROS (see properties), consequently can protect the skin from premature aging (wrinkles) and the occurrence of cancers. In contrast, infrared rays (IRs) do not seem to cause cancer, but they contribute significantly to skin aging, especially those in the IRA range (770–1400 nm) and increase the concentration of metalloproteinase-1 (MMP-1) in dermal fibroblasts of the matrix (Krutmann et al., 2012). MMP-1 is a zinc-dependent protease that degrades collagen and other extracellular matrix molecules and is a potential target in cancer therapy, indicating the great need to protect against IR radiation for future formulations. Advantageously, MMP-1 mRNA expression was reduced significantly in the presence of antioxidants (Grether-Beck et al., 2015). Thus, due to its high antiradical activity, the incorporation of pyomelanin in a cosmetic formulation could be beneficial for the protection against UV as well as IR radiation.

### As Material and Environmentally Friendly Protectant

Since pyomelanin absorbs light throughout the UV–visible spectra, it would also be effective as a glass or plastic tinting agent for eyeglasses, contact lenses, car and house windows, office buildings, etc. Likewise, such pigments would be effective agents in protecting industrial materials, the rate of deterioration of paint, wood, plastic, and rubber being dramatically increased by exposure to UV radiation. On the other hand, pyomelanin could serve as an antifouling agent. Indeed, it significantly reduces larval attachment and metamorphosis in the mussel *Mytilus coruscus*, hence preventing the attachment of fouling organisms particularly on boat hulls (Zeng et al., 2017). At least, pyomelanin chelates heavy metals, for instance, uranium present in some contaminated soils (Turick et al., 2008a, 2008b, 2010), as other melanin does.

### As a Promising polymer for Bioelectronics and Energy

Melanins generally have some energy conversion ability, conductivity, and semiconductor properties. They collect energy from lower-energy radiation sources, and kick electrons into excited states, initiating a process that would end up producing chemical energy. Interestingly, the freestanding film of melanin electrochemically obtained from L-Dopa exhibited photoconductivity (Subianto et al., 2005). To explain this effect, it was demonstrated that (i) the increase in the electron-transfer properties of melanin is independent of the energy of the incident photons; (ii) melanin functions as a transducer, oxidizing water, pushing apart water molecules, as well as recruiting back ions into molecules that are subsequently polarized again; and (iii) melanin drives the photon energy of lower-energy radiation sources by quenching electrons and initiating an ionic event independently of their relative energy contention (Solis et al., 2007). The same mechanism can be predicted for pyomelanin that exhibits an electrochemical response after UV-light exposition (see properties). The HGA pigment should thus be developed as a component for solar panel energy converters, with better advantages due to its high thermo- and photostability as well as highly protectant-material properties.

Pyomelanin has an important redox cycling nature with multiple quinone moieties, and the discharge or recharge of electrons from quinone centers is likely followed by the repartition of electrons and protons within the polymer until equilibrium is reached. Pyomelanin can thus conduct electricity like an electronic-ionic hybrid conductor for bioelectronic applications by only femtograms per cell (Turick et al., 2010), better than melanin. The HGA pigment has a great ability for electricity storage and could replace heavy metals in the construction of long-life, deep cycle, and rechargeable batteries of a new generation. Indeed, because most redox transformations involving melanin occur at low redox potential (*E* < 1–2 V), the variation in the energy level associated with electron exchanges is small relative to the strength of the covalent bonds, which hold the quinone structure together. Henceforth, pyomelanin should be incorporated into batteries that might be charged and discharged numerous times without affecting their stability. To replace the electron substrate with a heavy metal, a recent method for producing Li-pyomelanin (LPM), Na-pyomelanin (SPM), and K-pyomelanin (PPM) has been described as well as the related safety batteries (Popa et al., 2019). LPM, SPM, or PPM was placed in the negative compartment (anode), which led to the decreased tendencies to overheat and/or explode. Such battery developments deserve further investigations, especially using various molecular sizes of pyomelanin.

## Conclusions

In this updated review, all microbial pathways, including regulation-inhibition of pyomelanin synthesis and structural data, have been extensively covered. These methodologies would be useful to find microorganisms or enzymes more efficient for the production of the pigment. At this time, we conclude the amount of available pyomelanin, the prime limiting factor for all these years, is no longer an obstacle, offering realistic and functional applications in highly coveted fields and ensuring the fair rehabilitation of the polymer. The production has been resolved by either using laccase and starting from 2,5-DMPA or the overexpressed *Y. lipolytica* 4-HPPD recombinant enzyme cultivated with L-Tyr, two very recent and convenient processes that can be redimensioned and still optimized for an industrial unit.

## Supplementary Material

kuac013_Supplemental_File

## References

[bib1] Aarenstrup L. , Falch A.-M., Jakobsen K.K., Neve S., Henriksen L.O., Tommerup N., Leffers H., Kristiansen K. (2002). Expression and post-translational modification of human 4-hydroxy-phenylpyruvate dioxygenase. Cell Biology International 26(7), 615–625. 10.1006/cbir.2002.0896 12127941

[bib2] Abdul-Hussien Z.R. , Atia S.S. (2017). Antimicrobial effect of pyomelanin extracted from *Pseudomonas aeruginosa*. International Journal Development Research, 7, 8692. https://www.journalijdr.com/antimicrobial-effect-pyomelanin-extracted-pseudomonas-aeruginosa

[bib3] Adachi K.A. , Iwayama Y., Tanioka H., Takeda Y. (1966). Purification and properties of homogentisate oxygenase from *Pseudomonas fluorescens*. Biochimica et Biophysica Acta (BBA) - Enzymology and Biological Oxidation, 118(1), 88–97. 10.1016/S0926-6593(66)80147-9 5954067

[bib4] Ahmad S. , Lee S.Y., Kong H.G., Jo E.J., Choi H.K., Khan R., Lee S.W. (2016). Genetic determinants for pyomelanin production and its protective effect against oxidative stress in *Ralstonia solanacearum*. Plos One, 11(8), e0160845. 10.1371/journal.pone.0160845 27513990 PMC4981395

[bib7] Almeida-Paes R. , Almeida-Silva F., Pinto G.C.M., Almeida M.A., Muniz M.M., Pizzini C.V., Gerfen G.J., Nosanchuk J.D., Zancopé-Oliveira R.M. (2018). L-tyrosine induces the production of a pyomelanin-like pigment by the parasitic yeast-form of *Histoplasma capsulatum*. Medical Mycology, 56(4), 506–509. 10.1093/mmy/myx068 28992332 PMC6075583

[bib6] Almeida , -Paes R., Figueiredo-Carvalho M.H., Brito-Santos F., Almeida-Silva F., de Oliveira M.M.E., Zancopé-Oliveira R.M. (2016). Melanins protect *Sporothrix brasiliensis* and *Sporothrix schenckii* from the antifungal effects of Terbinafine. Plos One, 11(3), e0152796. 10.1371/journal.pone.0152796 27031728 PMC4816517

[bib5] Almeida-Paes R. , Frases S., Araújo G., de S., de Oliveira M.M.E., Gerfen G.J., Nosanchuk J.D., Zancope-Oliveira R.M. (2012). Biosynthesis and functions of a melanoid pigment produced by species of the *Sporothrix* complex in the presence of L-tyrosine. Applied and Environmental Microbiology, 78(24), 8623–8630. 10.1128/AEM.02414-12 23042177 PMC3502921

[bib8] Amouric A. , Liebgott P.-P., Brochier-Armanet C., Lorquin J. (2014). *Halomonas olivaria* sp. nov., a moderately halophilic bacterium isolated from olive-processing effluents. International Journal of Systematic and Evolutionary Microbiology, 64(Pt_1), 46–54. 10.1099/ijs.0.049007-0 24030688

[bib9] Anderson J.J. , Dagley S. (1980). Catabolism of aromatic acids in *Trichosporon cutaneum*. Journal of Bacteriology, 141(2), 534–543. https://jb.asm.org/content/141/2/534 7364712 10.1128/jb.141.2.534-543.1980 PMC293656

[bib10] Andrews S.C. , Robinson A.K., Rodriguez-Quiñones F. (2003). Bacterial iron homeostasis. FEMS Microbiology Reviews, 27(2-3), 215–237. 10.1016/S0168-6445(03)00055-X 12829269

[bib11] Apte M. , Girme G., Bankar A., RaviKumar A., Zinjarde S. (2013). 3,4-dihydroxy-L-phenylalanine-derived melanin from *Yarrowia lipolytica* mediates the synthesis of silver and gold nanostructures. Journal of Nanobiotechnology, 11(1), 2. 10.1186/1477-3155-11-2 23363424 PMC3660187

[bib12] Araújo M. , Viveiros R., Correia T.R., Correia I.J., Bonifácio V.D., Casimiro T., Aguiar-Ricardo A. (2014). Natural melanin: A potential pH-responsive drug release device. International Journal of Pharmaceutics, 469(1), 140–145. 10.1016/j.ijpharm.2014.04.051 24768404

[bib13] Aravindakshan I. , Haberman H.F. (1998). 33 lipomelanin sunscreen composition. Patent US 5,750,093. https://patents.google.com/patent/US5750093A

[bib14] Arcos M. , Olivera E.R., Arias S., Naharro G., Luengo J.M. (2010). The 3,4-dihydroxyphenylacetic acid catabolon, a catabolic unit for degradation of biogenic amines tyramine and dopamine in *Pseudomonas putida* U. Environmental Microbiology, 12, 1684–704. 10.1111/j.1462-2920.2010.02233.x 20482587

[bib15] Arias-Barrau E. , Olivera E.R., Luengo J.M., Fernández C., Galán B., García J.L., Díaz E., Miñambres B. (2004). The homogentisate pathway: A central catabolic pathway involved in the degradation of L-phenylalanine, L-tyrosine, and 3-hydroxyphenylacetate in *Pseudomonas putida*. Journal of Bacteriology, 186(15), 5062–5077. 10.1128/JB.186.15.5062-5077.2004 15262943 PMC451635

[bib16] Arias-Barrau E. , Sandoval A., Naharro G., Olivera E.R., Luengo J.M. (2005). A two-component hydroxylase involved in the assimilation of 3-hydroxyphenyl acetate in *Pseudomonas putida*. Journal of Biological Chemistry, 280(28), 26435–26447. 10.1074/jbc.M501988200 15866873

[bib17] Arnoux J.B. , Le Quan Sang K.H., Brassier A., Grisel C., Servais A., Wippf J., Dubois S., Sireau N., Job-Deslandre C., Ranganath L., De Lonlay P. (2015). Old treatments for new insights and strategies: Proposed management in adults and children with alkaptonuria. Journal of Inherited Metabolic Disease, 38(5), 791–796. 10.1007/s10545-015-9844-6 25860819

[bib18] Aubi O. , Flydal M.I., Zheng H., Skjærven L., Rekand I., Leiros H.-K.S., Haug B.E., Cianciotto N.P., Martinez A., Underhaug J. (2015). Discovery of a specific inhibitor of pyomelanin synthesis in *Legionella pneumophila*. Journal of Medicinal Chemistry, 58(21), 8402–8412. 10.1021/acs.jmedchem.5b01589 26458252

[bib19] Baggi G. , Boga M.M., Catelani D., Galli E., Treccani V. (1983). Styrene catabolism by a strain of *Pseudomonas fluorescens*. Systematic and Applied Microbiology, 4(1), 141–147. 10.1016/S0723-2020(83)80042-3 23196308

[bib21] Ben Tahar I. , Fickers P., Dziedzic A., Ploch D., Skora B., Kus-Liskiewicz M. (2019a). Green pyomelanin-mediated synthesis of gold nanoparticles: modeling and design, physic-chemical and biological characteristics. Microbial Cell Factories, 18(1), 210. 10.1186/s12934-019-1254-2 31796078 PMC6891958

[bib20] Ben Tahar I. , Kus-Liśkiewicz M., Lara Y., Javaux E., Fickers P. (2019b). Characterization of a non-toxic pyomelanin pigment produced by the yeast *Yarrowia lipolytica*. Biotechnology Progress, 36(2), e2912. 10.1002/btpr.2912 31525285

[bib22] Bergeron A. , D'Astous M., Timm D.E., Tanguay R.M. (2001). Structural and functional analysis of missense mutations in fumarylacetoacetate hydrolase, the gene deficient in hereditary tyrosinemia type 1. Journal of Biological Chemistry, 276(18), 15225–15231. 10.1074/jbc.M009341200 11278491

[bib23] Berka R.M. , Schneider P., Golightly E.J., Brown S.H., Madden M., Brown K.M., Halkier T., Mondorf K., Xu F. (1997). Characterization of the gene encoding an extracellular laccase of *Myceliophtora thermophila* and analysis of the recombinant enzyme expressed in *Aspergillus oryzae*. Applied and Environmental Microbiology, 63(8), 3151–3157. 10.1128/aem.63.8.3151-3157.1997 9251203 PMC168614

[bib24] Bettinger C.J. , Bruggeman J.P., Misra A., Borenstein J.T., Langer R. (2009). Biocompatibility of biodegradable semiconducting melanin films for nerve tissue engineering. Biomaterials, 30(17), 3050–3057. 10.1016/j.biomaterials.2009.02.018 19286252 PMC4059055

[bib25] Blakley E.R. (1977). The catabolism of L-tyrosine by an *Arthrobacter* sp. Canadian Journal of Microbiology, 23(9), 1128–1139. 10.1139/m77-169 20216

[bib26] Bloomer J.L. , Damodaran K.M. (1980). An efficient synthesis of homogentisic acid. Synthesis, 2(02), 111. 10.1055/s-1980-28931

[bib27] Boles B.R. , Singh P.K. (2008). Endogenous oxidative stress produces diversity and adaptability in biofilm communities. Proceedings of the National Academy of Sciences, 105(34), 12503–12508. 10.1073/pnas.0801499105 PMC2527941 18719125

[bib28] Bolognese F. , Scanferla C., Caruso E., Orlandi V.T. (2019). Bacterial melanin production by heterologous expression of 4-hydroxyphenylpyruvate dioxygenase from *Pseudomonas aeruginosa*. International Journal of Biological Macromolecules, 133, 1072–1080. 10.1016/j.ijbiomac.2019.04.061 31029629

[bib29] Borowski T. , Bassan A., Siegbahn P.E.M. (2004). 4-hydroxyphenylpyruvate dioxygenase: A hybrid density functional study of the catalytic reaction mechanism. Biochemistry, 43(38), 12331–12342. 10.1021/bi049503y 15379572

[bib30] Borowski T. , Georgiev V., Siegbahn P.E.M. (2005). Catalytic reaction mechanism of homogentisate dioxygenase: A hybrid DFT study. Journal of the American Chemical Society, 127(49), 17303–17314. 10.1021/ja054433j 16332080

[bib31] Bostock S. , Renfrew A.H. (1978). An improved synthesis of homogentisic acid and its lactone. Synthesis, 1978(01), 66–67. 10.1055/s-1978-24680

[bib32] Carreira A. , Ferreira L.M., Loureiro V. (2001). Production of brown tyrosine pigments by the yeast *Yarrowia lipolytica*. Journal of Applied Microbiology, 90(3), 372–379. 10.1046/j.1365-2672.2001.01256.x 11298232

[bib33] Chang T.-S. (2009). An updated review of tyrosinase inhibitors. International Journal of Molecular Sciences, 10(6), 2440–2475. 10.3390/ijms10062440</bib 19582213 PMC2705500

[bib34] Chapman P.J. , Dagley S. (1962). Oxidation of homogentisate acid by cell-free extracts of a *Vibrio*. Journal of General Microbiology, 28(2), 251–256. 10.1099/00221287-28-2-251 13878198

[bib35] Coon S.L. , Kotob S., Jarvis B.B., Wang S., Fuqua W.C., Weiner R.M. (1994). Homogentisic acid is the product of *mel*A, which mediates melanogenesis in the marine bacterium *Shewanella colwelliana*. Applied and Environmental Microbiology, 60(8), 3006–3010. 10.1128/AEM.60.8.3006-3010.1994 8085836 PMC201756

[bib36] Couto S.R. , Toca J.L. (2006). Inhibitors of laccases: A review. Current Enzyme Inhibition, 2(4), 343–352. 10.2174/157340806778699262

[bib37] Crawford R.L. (1975). Degradation of 3-hydroxybenzoate by bacteria of the genus *Bacillus*. Applied Microbiology, 30(3), 439–444. https://pubmed.ncbi.nlm.nih.gov/810087/. 810087 10.1128/am.30.3.439-444.1975 PMC187200

[bib39] Crawford R.L. (1976). Degradation of homogentisate by strains of *Bacillus and Morexella*. Canadian Journal of Microbiology, 22(2), 276–280. 10.1139/m76-037 1260531

[bib38] Crawford RL , Hutton SW, Chapman PJ. (1975). Purification and properties of gentisate 1,2-dioxygenase from *Moraxella osloensis*. Journal of Bacteriology, 121(3), 794–799. 10.1128/jb.121.3.794-799.1975 234947 PMC246005

[bib40] Cuskey S.M. , Olsen R.H. (1988). Catabolism of aromatic biogenic amines by *Pseudomonas aeruginosa* PAO1 via *meta* cleavage of homoprotocatechuic acid. Journal of Bacteriology, 170(1), 393–399. 10.1128/jb.170.1.393-399.1988 3121590 PMC210655

[bib41] David C. , Daro A., Szalai E., Atarhouch T., Mergeay M. (1996). Formation of polymeric pigments in the presence of bacteria and comparison with chemical oxidative coupling. II. Catabolism of tyrosine and hydroxyphenylacetic acid by *Alcaligenes eutrophus* CH34 and mutants. European Polymer Journal, 32(6), 669. 10.1016/0014-3057(95)00207-3

[bib42] Dawley R.M. , Flurkey W.H. (1993). 4-hexylresorcinol, a potent inhibitor of mushroom tyrosinase. Journal of Food Science, 58(3), 609–610. 10.1111/j.1365-2621.1993.tb04336.x

[bib43] Deforrest Abbott Jr L. , Doyle-Smith J. (1949). Chemical preparation of homogentisic acid. Journal of Biological Chemistry, 179(1), 365–368. https://pubmed.ncbi.nlm.nih.gov/18119250/18119250

[bib44] Epstein H. , Manga P., Koshoffer A., Story D., Simion T., Boissy R. (2007). Does melanin have an SPF and can it be measured? Journal of Cosmetic Science, 58, 596–597. https://library.scconline.org/v058n05/107

[bib45] Eslami M. , Zare H.R., Namazian M. (2014). The effect of solvents on the electrochemical behavior of homogentisic acid. Journal of Electroanalytical Chemistry, 720-721, 76–83. 10.1016/j.jelechem.2014.03.010

[bib46] Fang W. , Fernandes E.K.K., Roberts D.W., Bidochka M.J., St Leger R.J. (2010). A laccase exclusively expressed by *Metarhizium anisopliae* during isotropic growth is involved in pigmentation, tolerance to abiotic stresses and virulence. Fungal Genetics and Biology, 47(7), 602–607. 10.1016/j.fgb.2010.03.011 20382249

[bib47] Fernández-Cañon J.M. , Peñalva M.A. (1995). Molecular characterization of a gene encoding a homogentisate dioxygenase from *Aspergillus nidulans* and identification of its human and plant homologues. Journal of Biological Chemistry, 270(36), 21199–21205. 10.1074/jbc.270.36.21199 7673153

[bib48] Fernandez-Cañon J.M. , Peñalva M.A. (1997). Spectrophotometric determination of homogentisate using *Aspergillus nidulans* homogentisate dioxygenase. Analytical Biochemistry, 245(2), 218–221. 10.1006/abio.1996.9957 9056215

[bib49] Fernández-Cañon J.M. , Baetscher M.W., Finegold M., Burlingame T., Gibson K.M., Grompe M. (2002). Maleylacetoacetate isomerase (MAAI/GSTZ)-deficient mice reveal a glutathione-dependent nonenzymatic bypass in tyrosine catabolism. Molecular and Cellular Biology, 22(13), 4943–4951. 10.1128/mcb.22.13.4943-4951.2002 12052898 PMC133921

[bib50] Ferrer-Sevillano F. , Fernández-Cañon J.M. (2007). Novel *phac*B-encoded cytochrome P450 monooxygenase from *Aspergillus nidulans* with 3-hydroxyphenylacetate 6-hydroxylase and 3,4-dihydroxyphenylacetate 6-hydroxylase activities. Eukaryotic Cell, 6(3), 514–520. 10.1128/ec.00226-06 17189487 PMC1828918

[bib51] Frases S. , Salazar A., Dadachova E., Casadevall A. (2007). *Cryptococcus neoformans* can utilize the bacterial melanin precursor homogentisic acid for fungal melanogenesis. Applied and Environmental Microbiology, 73(2), 615–621. 10.1128/aem.01947-06 17098915 PMC1796974

[bib52] Fuqua W.C. , Coyne V.E., Stein D.C., Lin C.-M., Weiner R.M. (1991). Characterization of *mel*A: A gene encoding melanin biosynthesis from the marine bacterium *Shewanella colwelliana*. Gene, 109(1), 131–136. 10.1016/0378-1119(91)90598-6 1756973

[bib53] Ganesh Kumar C. , Mongolla P., Pombala S., Kamle A., Joseph J. (2011). Physicochemical characterization and antioxidant activity of melanin from a novel strain of *Aspergillus bridgeri* ICTF-201. Letters in Applied Microbiology, 53(3), 350–358. 10.1111/j.1472-765x.2011.03116.x 21726247

[bib54] Ganesh Kumar C. , Sahu N., Narender Reddy G., Prasad R.B.N., Nagesh N., Kamal A. (2013). Production of melanin pigment from *Pseudomonas stutzeri* isolated from red seaweed *Hypnea musciformis*. Letters in Applied Microbiology, 57(4), 295–302. 10.1111/lam.12111 23725061

[bib55] Garcia-Rivera J. , Eisenman H.C., Nosanchuk J.D., Aisen P., Zaragoza O., Moadel T., Dadachova E., Casadevall A. (2005). Comparative analysis of *Cryptococcus neoformans* acid-resistant particles generated from pigmented cells grown in different laccase substrates. Fungal Genetics and Biology, 42(12), 989–998. 10.1016/j.fgb.2005.09.003 16289955

[bib56] Gibello A. , Ferrer E., Sanz J., Martin M. (1995). Polymer production by *Klebsiella pneumoniae* 4-hydroxyphenylacetic acid hydroxylase genes cloned in *Escherichia coli*. Applied and Environmental Microbiology, 61(12), 4167–4171. 10.1128/aem.61.12.4167-4171.1995 8534083 PMC167727

[bib57] Grether-Beck S. , Marini A., Jaenicke T., Krutmann J. (2015). Effective photoprotection of human skin against infrared A radiation by topically applied antioxidants: Results from a vehicle controlled, double-blind, randomized study. Photochemistry and Photobiology, 91(1), 248–250. 10.1111/php.12375 25349107

[bib58] Guo J. , Rao Z., Yang T., Man Z., Xu M., Zhang X., Yang S.T. (2015). Cloning and identification of a novel tyrosinase and its overexpression in *Streptomyces kathirae* SC-1 for enhancing melanin production. FEMS Microbiology Letters, 362(8), 1–7. 10.1093/femsle/fnv041 25761752

[bib59] Hareland W.A. , Crawford R.L., Chapman P.J., Dagley S. (1975). Metabolic function and properties of 4-hydroxyphenylacetic acid 1-hydroxylase from *Pseudomonas acidovorans*. Journal of Bacteriology, 121(1), 272–285. 10.1128/jb.121.1.272-285.1975 234937 PMC285641

[bib60] Hegedus Z.L. , Nayak U. (1994). Homogentisic acid and structurally related compounds as intermediates in plasma soluble melanin formation and in tissue toxicities. Archives Internationales de Physiologie, de Biochimie et de Biophysique, 102(3), 175–181. 10.3109/13813459409007534 8000039

[bib61] Heinekamp T. , Thywißen A., Macheleidt J., Keller S., Valiante V., Brakhage A.A. (2013). *Aspergillus fumigatus* melanins: Interference with the host endocytosis pathway and impact on virulence. Frontiers in Microbiology, 3, 440. 10.3389/fmicb.2012.00440 23346079 PMC3548413

[bib62] Hiraku Y. , Yamasaki M., Kawanishi S. (1998). Oxidative DNA damage induced by homogentisic acid, a tyrosine metabolite. FEBS Letters, 432(1-2)), 13–16. 10.1016/S0014-5793(98)00823-0 9710241

[bib63] Hudecová S. , Straková S., Krizanova O. (1995). Purification of the homogentisic acid oxidase from mammalian liver. The International Journal of Biochemistry & Cell Biology, 27(12), 1357–1363. 10.1016/1357-2725(95)00091-3 8581831

[bib64] d'Ischia M. , Wakamatsu K., Napolitano A., Briganti S., Garcia-Borron J.-C., Kovacs D., Meredith P., Pezzella A., Picardo M., Sarna T., Simon J.D., Ito S. (2013). Melanins and melanogenesis: Methods, standards, protocols. Pigment Cell & Melanoma Research, 26(5), 616–633. 10.1111/pcmr.12121 23710556

[bib65] Janocha S. , Wolz W., Srsen S., Srsnova K., Montagutelli X., Guénet J.L., Grimm T., Kress W., Müller C.R. (1994). The human gene for alkaptonuria (AKU) maps to chromosome 3q. Genomics, 19(1), 5–8. 10.1006/geno.1994.1003 8188241

[bib66] Keith K.E. , Killip L., He P.Q., Moran G.R., Valvano M.A. (2007). *Burkholderia cenocepacia* C5424 produces a pigment with antioxidant properties using a homogentisate intermediate. Journal of Bacteriology, 189(24), 9057–9065. 10.1128/JB.00436-07 17933889 PMC2168628

[bib67] Kelly C.J. , Johnson T.C. (1978). Effects of *p*-chlorophenylalanine and α-methylphenylalanine on amino acid uptake and protein synthesis in mouse neuroblastoma cells. Biochemical Journal, 174(3), 931–938. 10.1042/bj1740931 153135 PMC1185998

[bib68] Ketelboeter L.M. , Potharla V.Y., Sonia L., Bardy S.L. (2014). NTBC treatment of the pyomelanogenic *Pseudomonas aeruginosa* clinical isolate PA1111 inhibits pigment production and increases sensitivity to oxidative stress. Current Microbiology, 69(3), 343–348. 10.1007/s00284-014-0593-9 24801336 PMC4113677

[bib69] Kotob S.I. , Coon S.L., Quintero E.J., Weiner R.M. (1995). Homogentisic acid is the primary precursor of melanin synthesis in *Vibrio cholerae*, a hyphomonas strain, and *Shewanella colwelliana*. Applied and Environmental Microbiology, 61(4), 1620–1622. 10.1128/aem.61.4.1620-1622.1995 7747976 PMC167418

[bib70] Krutmann J. , Morita A., Chung J.H. (2012). Sun exposure: What molecular photodermatology tells us about its good and bad sides. Journal of Investigative Dermatology, 132(3), 976–984. 10.1038/jid.2011.394 22170486

[bib71] Kurian N.K. , Bhat S.G. (2018). Data on the characterization of non-cytotoxic pyomelanin produced by marine *Pseudomonas stutzeri* BTCZ10 with cosmetological importance. Data in Brief, 18, 1889–1894. 10.1016/j.dib.2018.04.123 29904692 PMC5998704

[bib73] La Du B.N. , Zannoni V.G. (1963). Oxidation of homogentisic acid catalyzed by horseradish peroxidase. Biochimica et Biophysica Acta (BBA) - Specialized Section on Enzymological Subjects, 67, 281–287. 10.1016/0926-6569(63)90235-9

[bib72] La Du B.N. , Zannoni V.G., Laster L., Seegmiller J.E. (1958). The nature of the defect in tyrosine metabolism in alkaptonuria. Journal of Biological Chemistry, 230(1), 251–260. 10.1016/S0021-9258(18)70560-7 13502394

[bib74] Larroude M. , Nicaud J.-M., Rossignol T.A. (2021a). *Yarrowia lipolytica* chassis strains engineered to produce aromatic amino acids via the shikimate pathway. Microbial Biotechnology, 14(6), 2420–2434. 10.1111/1751-7915.13745 33438818 PMC8601196

[bib75] Larroude M. , Onésime D., Rué O., Nicaud J.-M., Rossignol T.A. (2021b). *Yarrowia lipolytica* strain engineered for pyomelanin production. Microorganisms, 9(4), 838. 10.3390/microorganisms9040838 33920006 PMC8071058

[bib76] Leaf G. , Neuberger A. (1948). The preparation of homogentisic acid and of 2:5-dihydroxyphenylethylamine. Biochemical Journal, 43(4), 606–610. 10.1042/bj0430606 16748459 PMC1274783

[bib77] Lederer B. , Böger P. (2005). Recombinant *p*-hydroxyphenylpyruvate dioxygenase of high activity. Zeitschrift fÃ¼r Naturforschung C, 60(7-8), 549–556. 10.1515/znc-2005-7-807 16163828

[bib78] Liaw S.J. , Lee Y.L., Hsueh P.R. (2010). Multidrug resistance in clinical isolates of *Stenotrophomonas maltophilia*: Roles of integrons, efflux pumps, phosphoglucomutase (SpgM), and melanin and biofilm formation. International Journal of Antimicrobial Agents, 35(2), 126–130. 10.1016/j.ijantimicag.2009.09.015</bib 19926255

[bib81] Liebgott P.-P. , Amouric A., Comte A., Tholozan J.-L., Lorquin J. (2009). Hydroxytyrosol from tyrosol using hydroxyphenylacetic acid-induced bacterial cultures and evidence on the role of 4-HPA 3-hydroxylase. Research in Microbiology 160(10), 757–766. 10.1016/j.resmic.2009.09.015 19837158

[bib80] Liebgott P.-P. , Labat M., Amouric A., Tholozan J.-L., Lorquin J. (2008). Tyrosol degradation *via* the homogentisic acid pathway in a newly isolated *Halomonas* strain from olive processing effluents. Journal of Applied Microbiology 105(6), 2084–2095. 10.1111/j.1365-2672.2008.03925.x 19120654

[bib79] Liebgott P.-P. , Labat M., Casalot L., Amouric A., Lorquin J. (2007). Bioconversion of tyrosol into hydroxytyrosol and 3,4-dihydroxyphenylacetic acid under hypersaline conditions by a new *Halomonas* sp. strain HTB24. FEMS Microbiology Letters, 276(1), 26–33. 10.1111/j.1574-6968.2007.00896.x 17937662

[bib82] Lorquin F. , Ziarelli F., Amouric A., Di Giorgio C., Robin M., Piccerelle P., Lorquin J. (2021). Production and properties of non-cytotoxic pyomelanin by laccase and comparison to bacterial and synthetic pigments. Scientific Reports, 11(1), 8538. 10.1038/s41598-021-87328-2 33879803 PMC8058095

[bib90] McKiernan P. (2006). Nitisinone in the treatment of hereditary tyrosinaemia type 1. Drugs, 66(6), 743–750. 10.2165/00003495-200666060-00002 16706549

[bib83] Maccarrone M. , Catani M.V., Iraci S., Melino G., Finazzi Agrò A. (1997). A survey of reactive oxygen species and their role in dermatology. Journal of the European Academy of Dermatology and Venereology, 8(3), 185–202. 10.1016/S0926-9959(97)00076-7

[bib84] Magarelli M. , Passamonti P., Renieri C. (2010). Purification, characterization and analysis of sepia melanin from commercial sepia ink (*Sepia officinalis*). Revista CES Medicina Veterinaria y Zootecnia, 5, 18–28. https://revistas.ces.edu.co/index.php/mvz/article/view/1424

[bib85] Mahmood H.M. , Mohammed A.K., Fleih M.T. (2016). Cytotoxic effect of pyomelanin pigment produced from local *Pseudomonas aeruginosa* isolates on different cell lines using MTT assay. Iraqi Journal of Biotechnology, 15, 46–52. https://www.researchgate.net/publication/301593914

[bib86] Martin Jr J.P. , Batkoff B. (1987). Homogentisic acid autoxidation and oxygen radical generation: Implications for the etiology of alkaptonuric arthritis. Free Radical Biology and Medicine, 3(4), 241–250. 10.1016/S0891-5849(87)80031-X 3121448

[bib87] Martínez L.M. , Martinez A., Gosset G. (2019). Production of melanins with recombinant microorganisms. Frontiers in Bioengineering and Biotechnology, 7, 285. 10.3389/fbioe.2019.00285 31709247 PMC6821874

[bib88] Martínez-Sotres C. , López-Albarrán P., Cruz-de-León J., García-Moreno T., Rutiaga-Quiñones J.G., Vázquez-Marrufo G., Tamariz-Mascarúa J., Herrera-Bucio R. (2012). Medicarpin, an antifungal compound identified in hexane extract of *Dalbergia congestiflora* Pittier heartwood. International Biodeterioration & Biodegradation, 69, 38–40. 10.1016/j.ibiod.2011.11.016

[bib89] Martínez-Sotres C. , Rutiaga-Quiñones J.G., Herrera-Bucio R., Gallo M., López-Albarrán P. (2015). Molecular docking insights into the inhibition of laccase activity by medicarpin. Wood Science and Technology, 49(4), 857–868. 10.1007/s00226-015-0734-8

[bib91] Mefford I.N. , Kincl L., Dykstra K.H., Simpson J.T., Markey S.P., Dietz S., Wightman R.M. (1996). Facile oxidative decarboxylation of 3,4-dihydroxyphenylacetic acid catalyzed by copper and manganese ions. Biochimica et Biophysica Acta (BBA) - General Subjects, 1290(3), 224–230. 10.1016/0304-4165(96)00017-7 8765124

[bib92] Mekala L.P. , Mohammed M., Chinthalapati S., Chinthalapati V.R. (2019). Pyomelanin production: Insights into the incomplete aerobic L-phenylalanine catabolism of a photosynthetic bacterium, *Rubrivivax benzoatilyticus* JA2. International Journal of Biological Macromolecules, 126, 755–764. 10.1016/j.ijbiomac.2018.12.142 30572055

[bib93] Mendez V. , Agullo L., Gonzalez M., Seeger M. (2011). The homogentisate and homoprotocatechuate central pathways are involved in 3- and 4-hydroxyphenylacetate degradation by *Burkholderia xenovorans* LB400. Plos One, 6(3), e17583. 10.1371/journal.pone.0017583 21423751 PMC3053370

[bib94] Mohamed M.E.S. , Ismail W., Heider J., Fuchs G. (2002). Aerobic metabolism of phenylacetic acids in *Azoarcus evansii*. Archives of Microbiology, 178(3), 180–192. 10.1007/s00203-002-0438-y 12189419

[bib95] Narayanan S. , Kurian N.K., Bhat S.G. (2020). Ultra-small pyomelanin nanogranules abiotically derived from bacteria-secreted homogentisic acid show potential applications in inflammation and cancer. BioNanoScience, 10(1), 191–203. 10.1007/s12668-019-00689-x

[bib96] Ndikuryayo F. , Moosavi B., Yang W.-C., Yang G.-F. (2017). 4‑hydroxyphenylpyruvate dioxygenase inhibitors: From chemical biology to agrochemicals. Journal of Agricultural and Food Chemistry, 65(39), 8523–8537. 10.1021/acs.jafc.7b03851 28903556

[bib97] Neuckermans J. , Mertens A., De Win D., Schwaneberg U., De Kock J. (2019). A robust bacterial assay for high-throughput screening of human 4-hydroxyphenylpyruvate dioxygenase inhibitors. Scientific Reports, 9(1), 14145. 10.1038/s41598-019-50533-1 31578365 PMC6775094

[bib98] Nikodinovic-Runic J. , Martin L.B., Babu R., Blau W., O'Connor K.E. (2009). Characterization of melanin-overproducing transposon mutants of *Pseudomonas putida* F6. Fems Microbiology Letters, 298, 174–183. 10.1111/j.1574-6968.2009.01716.x(2009). 19624744

[bib99] Nofsinger J. , Forest S., Simon J. (1999). Explanation for the disparity among absorption and action spectra of eumelanin. The Journal of Physical Chemistry B, 103(51), 11428–11432. 10.1021/jp992640y

[bib100] Nosanchuk J.D. , Casadevall A. (2003). The contribution of melanin to microbial pathogenesis. Cellular Microbiology, 5(4), 203–223. 10.1046/j.1462-5814.2003.00268.x 12675679

[bib101] Paliwal V. , Raju S.C., Modak A., Phale P.S., Purohit H.J. (2014). *Pseudomonas putida* CSV86: A candidate genome for genetic bioaugmentation. Plos One, 9(1), e84000. 10.1371/journal.pone.0084000 24475028 PMC3901652

[bib102] Pawelek J. , Osber M.P., Orlow S.J. (1995). Synthetic melanin as a sunscreen and tanning agent. Patent US5384116A. https://patents.google.com/patent/US5384116/pt

[bib103] Pfuhler S. , Fautz R., Ouedraogo G., Latil A., Kenny J., Moore C., Diembeck W., Hewitt N.J., Reisinger K., Barroso J. (2014). The cosmetics Europe strategy for animal-free genotoxicit testing: project status up-date. Toxicology in Vitro, 28(1), 18–23. 10.1016/j.tiv.2013.06.004 23811264

[bib104] Popa R. , Nealson K.H. (2014). Methods for producing melanin and inorganic fertilizer from fermentation leachates. Patent US2014/0360237A1. https://patents.google.com/patent/US20140360237A1/en

[bib105] Popa R. , Reseda C.A., Nealson K.H., Cimpoiasu V.M. (2019). Method for producing lithium-ion, sodium-ion and potassium-ion batteries with increased safety. Patent US2019/0058193A1. https://patents.google.com/patent/WO2017062550A1/en

[bib106] Pralea I.-E. , Moldovan R.-C., Petrache A.-M., Ilies M., Heghes S.-C., Ielciu I., Nicoară R., Moldovan M., Ene M., Radu M., Uifălean A., Iuga C.-A. (2019). From extraction to advanced analytical methods: The challenges of melanin analysis. International Journal of Molecular Sciences, 20(16), 3943. 10.3390/ijms20163943 PMC6719904 31412656

[bib107] Prasad C.S.N. , Adapa S.R. (1992). Synthesis of homogentisic acid by carbonylation. Indian Journal of Chemistry, 31B, 626–627. https://chemistry.mdma.ch/hiveboard/rhodium/pdf/homogentisic.pdf

[bib108] Premi S. , Wallisch S., Mano C.M., Weiner A.B., Bacchiocchi A., Wakamatsu K., Bechara E.J.H., Halaban R., Douki T., Brash D.E. (2015). Chemiexcitation of melanin derivatives induces DNA photoproducts long after UV exposure. Science, 347(6224), 842–847. 10.1126/science.1256022 25700512 PMC4432913

[bib109] Prieto M.A. , Garcia J.L. (1994). Molecular characterization of 4-hydroxyphenylacetate-3-hydroxylase of *Escherichia coli*, a two-protein component enzyme. Journal of Biological Chemistry, 269(36), 22823–22829. https://pubmed.ncbi.nlm.nih.gov/8077235/8077235

[bib110] Raspail C. , Graindorge M., Moreau Y., Crouzy S., Lefèbvre B., Robin A.Y., Dumas R., Matringe M. (2011). 4-hydroxyphenylpyruvate dioxygenase catalysis. Identification of catalytic residues and production of a hydroxylated intermediate shared with a structurally unrelated enzyme. Journal of Biological Chemistry, 286(29), 26061–26070. 10.1074/jbc.m111.227595 21613226 PMC3138293

[bib111] Ribera J. , Panzarasa G., Stobbe A., Osypova A., Rupper P., Klose D., Schwarze F.W.M.R. (2019). Scalable biosynthesis of melanin by the basidiomycete *Armillaria cepistipes*. Journal of Agricultural and Food Chemistry, 67(1), 132–139. 10.1021/acs.jafc.8b05071 30541276

[bib112] Roberts N.B. , Curtis S.A., Milan A.M., Ranganath L.M. (2015). The pigment in alkaptonuria relationship to melanin and other coloured substances: A review of metabolism, composition, and chemical analysis. JIMD Reports, 24, 51–66. https://dx.doi.org/10.1007%2F8904_2015_453 26093627 10.1007/8904_2015_453 PMC4582024

[bib113] Rodríguez-Rojas A. , Mena A., Martín S., Borrell N., Oliver A., Blázquez J. (2009). Inactivation of the *hmg*A gene of *Pseudomonas aeruginosa* leads to pyomelanin hyperproduction, stress resistance and increased persistence in chronic lung infection. Microbiology (Reading, England), 155(4), 1050–1057. 10.1099/mic.0.024745-0 19332807

[bib114] Ruzafa C. , Sanchez-Amat A., Solano F. (1995). Characterization of the melanogenic system in *Vibrio cholerae*, ATCC 14035. Pigment Cell Research, 8(3), 147–152. 10.1111/j.1600-0749.1995.tb00656.x 7567791

[bib115] Santucci A. , Bernardini G., Braconi D., Petricci E., Manetti F. (2017). 4Hydroxyphenylpyruvate dioxygenase and its inhibition in plants and animals: Small molecules as herbicides and agents for the treatment of human inherited diseases. Journal of Medicinal Chemistry, 60(10), 4101–4125. 10.1021/acs.jmedchem.6b01395 28128559

[bib116] Sapmak A. , Boyce K.J., Andrianopoulos A., Vanittanakom N. (2015). The *pbr*B gene encodes a laccase required for DHN-melanin synthesis in conidia of *Talaromyces* (*Penicillium*) *marneffei*. Plos One, 10(4), e0122728. 10.1371/journal.pone.0122728 25866870 PMC4395095

[bib117] Sarna T. , Swartz H.M. (2006). The physical properties of melanins. In J.J., Nordlund et al. (Eds.), The Pigmentary System, (2nd ed., pp. 311–341). Oxford University Press. 10.1002/9780470987100.ch16

[bib118] Schmaler-Ripcke J. , Sugareva V., Gebhardt P., Winkler R., Kniemeyer O., Heinekamp T., Brakhage A.A. (2009). Production of pyomelanin, a second type of melanin, via the tyrosine degradation pathway in *Aspergillus fumigatus*. Applied and Environmental Microbiology, 75(2), 493–503. 10.1128/AEM.02077-08 19028908 PMC2620705

[bib119] Schmidt S.R. , Muller C.R., Kress W. (1995). Murine liver homogentisate 1,2-dioxygenase. Purification to homogeneity and novel biochemical properties. European Journal of Biochemistry, 228(2), 425–430. 10.1111/j.1432-1033.1995.0425n.x 7705358

[bib120] Seo D. , Choi K.Y. (2020). Heterologous production of pyomelanin biopolymer using 4-hydroxyphenylpyruvate dioxygenase isolated from *Ralstonia pickettii* in *Escherichia coli*. Biochemical Engineering Journal, 157, 107548. 10.1016/j.bej.2020.107548

[bib121] Sigoillot C. , Record E., Belle V., Robert J.L., Levasseur A., Punt P.J., van den Hondel C.A.M.J.J., Fournel A., Sigoillot J.C., Asther M. (2004). Natural and recombinant fungal laccases for paper pulp bleaching. Applied Microbiology and Biotechnology, 64(3), 346–352. 10.1007/s00253-003-1468-3 14600793

[bib122] Singh D. , Kumar J., Kumar A. (2018). Isolation of pyomelanin from bacteria and evidence showing its synthesis by 4-hydroxyphenylpyruvate dioxygenase enzyme encoded by HPPD gene. International Journal of Biological Macromolecules, 119, 864–873. 10.1016/j.ijbiomac.2018.08.003 30081124

[bib123] Solis A. , Lara M., Rendon L. (2007). Photoelectrochemical properties of melanin. Nature Precedings, 2007. 10.1038/npre.2007.1312.1

[bib124] Staudenmaier H.R. , Hauer B., Ladner W., Mueller U., Pressler U., Meyer J. (1999). Fermentative preparation of 2,5-dihydroxyphenylacetic acid with *Beauveria*. Patent US5,955,328. https://patents.google.com/patent/US5955328

[bib125] Steinert M. , Engelhard H., Flugel M., Wintermeyer E., Hacker J. (1995). The LLY protein protects *Legionella pneumophila* from light but does not directly influence its intracellular survival in *Hartmannella vermiformis*. Applied and Environmental Microbiology, 61(6), 2428–2430. 10.1128/aem.61.6.2428-2430.1995 7793965 PMC167516

[bib126] Stoner R. , Blivaiss B.B. (1967). Reaction of quinone of homogentisic acid with biological amines. Arthritis & Rheumatism, 10(1), 53–60. 10.1002/art.1780100108 6019345

[bib127] Subianto S. , Will G., Meredith P. (2005). Electrochemical synthesis of melanin free-standing films. Polymer, 46(25), 11505–11509. 10.1016/j.polymer.2005.10.068

[bib128] Suemori A. , Nakajima K., Kurane R., Nakamura Y. (1996). Purification and characterization of *o*-hydroxyphenylacetate 5-hydroxylase, *m*-hydroxyphenylacetate 6-hydroxylase and *p*-hydroxyphenylacetate 1-hydroxylase from *Rhodococcus erythropolis*. Journal of Fermentation and Bioengineering, 81(2), 133–137. 10.1016/0922-338X(96)87590-8

[bib129] Sugumaran M. , Vaidyanathan C.S. (1978). Affinity chromatography of homogentisate-1,2-dioxygenase from *Aspergillus niger*. FEMS Microbiology Letters, 4(6), 343–347. 10.1111/j.1574-6968.1978.tb02895.x

[bib130] Sun X. , Bai R., Zhang Y., Wang Q., Fan X., Yuan J., Cui L., Wang P. (2013). Laccase-catalyzed oxidative polymerization of phenolic compounds. Applied Biochemistry and Biotechnology, 171(7), 1673–1680. 10.1007/s12010-013-0463-0 23996120

[bib131] Szewczyk G. , Zadlo A., Sarna M., Ito S., Wakamatsu K., Sarna T. (2016). Aerobic photoreactivity of synthetic eumelanins and pheomelanins: Generation of singlet oxygen and superoxide anion. Pigment Cell & Melanoma Research, 29(6), 669–678. 10.1111/pcmr.12514 27505632

[bib132] Tan T.-T. , Zhang X.-D., Miaoa Z., Yua Y., Dua S.-L., Houa X.-Y., Caia J. (2019). A single point mutation in *hmg*A leads to melanin accumulation in *Bacillus thuringiensis* BMB181. Enzyme and Microbial Technology, 120, 91–97. 10.1016/j.enzmictec.2018.10.007 30396405

[bib133] Titus G.P. , Mueller H.A., Burgner J., de Córdoba S.R., Peñalva M.A., Timm D.E. (2000). Crystal structure of human homogentisate dioxygenase. Nature Structural Biology, 7(7), 542–546. 10.1038/76756 10876237

[bib134] Tokuhara Y. , Shukuya K., Tanaka M., Sogabe K., Ejima Y., Hosokawa S., Ohsaki H., Morinishi T., Hirakawa E., Yatomi Y., Shimosawa T. (2018). Absorbance measurements of oxidation of homogentisic acid accelerated by the addition of alkaline solution with sodium hypochlorite pentahydrate. Scientific Reports, 8(1), 11364. 10.1038/s41598-018-29769-w 30054539 PMC6063975

[bib135] Trias J. , Vinas M., Guinea J., Loren J.G. (1989). Brown pigmentation in *Serratia marcescens* cultures associated with tyrosine metabolism. Canadian Journal of Microbiology, 35(11), 1037–1042. 10.1139/m89-172 2692797

[bib140] Turick C.E. , Beliaev A.S., Zakrajsek B.A., Reardon C.L., Lowy D.A., Poppy T.E., Maloney A., Ekechukwu A.A. (2009). The role of 4-hydroxyphenylpyruvate dioxygenase in enhancement of solid-phase electron transfer by *Shewanella oneidensis* MR-1. FEMS Microbiology Ecology, 68(2), 223–225. 10.1111/j.1574-6941.2009.00670.x 19573203

[bib137] Turick C.E. , Caccavo Jr F., Tisa L.S. (2003). Electron transfer from *Shewanella Algae* BrY to hydrous ferric oxide is mediated by cell-associated melanin. FEMS Microbiology Letters, 220(1), 99. 10.1016/S0378-1097(03)00096-X 12644234

[bib139] Turick C.E. , Caccavo Jr F., Tisa L.S. (2008a). Pyomelanin is produced by *Shewanella algae* BrY and affected by exogenous iron. Canadian Journal of Microbiology, 54(4), 334–339. 10.1139/W08-014 18389008

[bib141] Turick C.E. , Knox A.S., Becnel J.M., Ekechukwu A.A., Milliken C.E. (2010). Properties and function of pyomelanin. In M., Elnashar(ed.), Biopolymers, (pp. 449–472) IntechOpen. https://doi:10.5772/10273

[bib138] Turick C.E. , Knox A.S., Leverette C.L., Kritzas Y.G. (2008b) *In-situ* uranium immobilization by microbial metabolites. Journal of Environmental Radioactivity, 99(6), 890–899. 10.1016/j.jenvrad.2007.11.020 18222573

[bib136] Turick C.E. , Tisa L.S., Caccavo Jr F. (2002). Melanin production and use as a soluble electron shuttle for Fe(III) oxide reduction and as a terminal electron acceptor by *Shewanella algae* BrY. Applied and Environmental Microbiology, 68(5), 2436–2444. 10.1128/aem.68.5.2436-2444.2002 11976119 PMC127558

[bib142] Upadhyay S. , Torres G., Lin X. (2013). Laccases involved in 1,8-dihydroxynaphthalene melanin biosynthesis in *Aspergillus fumigatus* are regulated by developmental factors and copper homeostasis. Eukaryotic Cell, 12(12), 1641–1652. 10.1128/EC.00217-13 24123270 PMC3889567

[bib143] van Almsick A. (2009). New HPPD inhibitors, a proven mode of action as a new hope to solve current weed problems. Outlook in Pest Management, 20, 27–30. 10.1564/20feb09

[bib144] Van den Tweel W.J.J. , Janssens R.J.J., de Bont J.A.M. (1986). Degradation of 4-hydroxyphenylacetate by *Xanthobacter* 124X; Physiological resemblance with other Gram-negative bacteria. Antonie Van Leeuwenhoek, 52(4), 309–318. 10.1007/bf00428642 3767351

[bib145] Van den Tweel W.J.J. , Smits J.P., de Bont J.A.M. (1988). Catabolism of DL-α-phenylhydracrylic, phenylacetic and 4-hydroxyphenylacetic acids *via* homogentisic acid in a *Flavobacterium* sp. Archives of Microbiology, 149(3), 207–213. 10.1007/BF00422006

[bib146] Vasanthakumar A. , DeAraujo A., Mazurek J., Schilling M., Mitchell R. (2015). Pyomelanin production in *Penicillium chrysogenum* is stimulated by L-tyrosine. Microbiology (Reading, England), 161(6), 1211–1218. 10.1099/mic.0.000030 25568259

[bib147] Veldhuizen E.J.A. , Vaillancourt F.H., Whiting C.J., Hsiao M.M.-Y., Gingras G., Xiao Y., Tanguay R.M., Boukouvalas J., Eltis L.D. (2005). Steady-state kinetics and inhibition of anaerobically purified human homogentisate 1,2-dioxygenase. Biochemical Journal, 386(2), 305–314. 10.1042/bj20041370 15479158 PMC1134795

[bib148] Vijayan V. , Jasmin C., Anas A., Kuttan S.P., Vinothkumar S., Subrayan P.P., Nair S. (2017). Sponge-associated bacteria produce non-cytotoxic melanin which protects animal cells from photo-toxicity. Applied Biochemistry and Biotechnology, 183(1), 396–411. 10.1007/s12010-017-2453-0 28315112

[bib149] Wakamatsu K. , Fujikawa K., Zucca F.A., Zecca L., Ito S. (2003). The structure of neuromelanin as studied by chemical degradative methods. Journal of Neurochemistry, 86(4), 1015–1023. 10.1046/j.1471-4159.2003.01917.x 12887698

[bib150] Williamson P.R. (1994). Biochemical and molecular characterization of the diphenol oxidase of *Cryptococcus neoformans*: Identification as a laccase. Journal of Bacteriology, 176(3), 656–664. 10.1128/jb.176.3.656-664.1994 8300520 PMC205102

[bib151] Wójcik A. , Broclawik E., Siegbahn P.E.M., Lundberg M., Moran G., Borowski T. (2014). Role of substrate positioning in the catalytic reaction of 4-hydroxyphenylpyruvate dioxygenase––A QM/MM study. Journal of the American Chemical Society, 136(41), 14472–14485. 10.1021/ja506378u 25157877

[bib152] Yabuuchi E. , Ohyama A. (1972). Characterization of “pyomelanin”-producing strains of *Pseudomonas aeruginosa*. International Journal of Systematic Bacteriology, 22(2), 53–64. 10.1099/00207713-22-2-53

[bib154] Zannoni V.G. , Lomtevas N., Goldfinger S. (1969). Oxidation of homogentisic acid to ochronotic pigment in connective tissue. Biochimica et Biophysica Acta (BBA) - General Subjects, 177(1), 94–105. 10.1016/0304-4165(69)90068-3 4976426

[bib155] Zeng Z. , Guo X.P., Cai X., Wang P., Li B., Yang J.L., Wang X. (2017). Pyomelanin from *Pseudoalteromonas lipolytica* reduces biofouling. Microbial Biotechnology, 10(6), 1718–1731. 10.1111/1751-7915.12773 28834245 PMC5658579

[bib156] Zheng H. , Chatfield C.H., Liles M.R., Cianciotto N.P. (2013). Secreted pyomelanin of *Legionella pneumophila* promotes bacterial iron uptake and growth under iron-limiting conditions. Infection and Immunity, 81(11), 4182–4191. 10.1128/iai.00858-13 23980114 PMC3811826

[bib157] Zink O. , Paget E., Rolland A., Sailland A., Freyssinet G. (2012). Herbicide-tolerant plants through bypassing metabolic pathway. Patent US8,124,846B2. https://patents.google.com/patent/US8124846B2/en(2012).

[bib158] Zughaier S.M. , Ryley H.C., Jackson S.K. (1999). A melanin pigment purified from an epidemic strain of *Burkholderia cepacia* attenuates monocyte respiratory burst activity by scavenging superoxide anion. Infection and Immunity, 67(2), 908–913. 10.1128/iai.67.2.908-913.1999 9916107 PMC96403

