# Supplementary material for: New insights and advances on pyomelanin production: from microbial synthesis to applications
Source: J Ind Microbiol Biotechnol. 2022 Jul 22;49(4):kuac013. doi: 10.1093/jimb/kuac013 (PMC9338888; doi:10.1093/jimb/kuac013)
Supplement: kuac013_Supplemental_File [file kuac013_supplemental_file.docx]

**SUPPLEMENTARY MATERIAL**

**New insights and advances on pyomelanin production: from microbial synthesis to applications**

**Faustine Lorquin^1,2^, Philippe Piccerelle^2^, Caroline Orneto^2^, Maxime Robin^2^, Jean Lorquin^1,*^**

^1^ Aix-Marseille Université, Mediterranean Institute of Oceanology (MIO), 163 avenue de Luminy, 13288 Marseille Cedex 9, France

^2^ Aix-Marseille Université, Mediterranean Institute of Marine and Terrestrial Biodiversity and Ecology (IMBE), 27 boulevard Jean Moulin, 13385 Marseille Cedex 5, France

**Biosynthesis of the other allomelanins**. While the synthesis of pyomelanin begins with L-Phe and L-Tyr (see Fig. 1), that of DHN-, THN-, GHB, and catechol-melanin starts from endogenous precursors, acetyl-CoA, PAP, and catechol (this figure). The biosynthetic pathways involve polyketide synthases (PKS) through the DHN pathway common in fungi and some bacteria, polyphenol oxidases (PPOs) in plants through the catechol oligomers, RppA, a PKS of type III, and P450-mel, an enzyme of the cytochrome P450 family in fungi to produce 1,4,6,7,9,12-hexahydroxyperylene-3,10-quinone (HPQ) that readily auto-polymerizes to generate HPQ-melanin, tyrosinases (T) through GHB-melanin formation from *p*-aminophenol and glutamate in *Agaricus* species, and laccase through HGA (pyomelanin). ***Abbreviations***: 1,8-DHN, 1,8-dihydroxynaphtalene; GHB, γ-glutaminyl-4-hydroxybenzene; GDHB, γ-glutaminyl-3,4-dihydroxybenzene; GBQ, γ-glutaminyl-3,4-dihydroxybenzoquinone; HIBQ, 2-hydroxy-*p*-iminobenzoquinone; HPQ, 1,4,6,7,9,12-hexahydroxyperylene-3,10-quinone; PAP, *p*-aminophenol; TAT, tyrosine transaminase; 1,3,6,8-THN, 1,3,6,8-tetrahydroxynaphtalene.

.

**
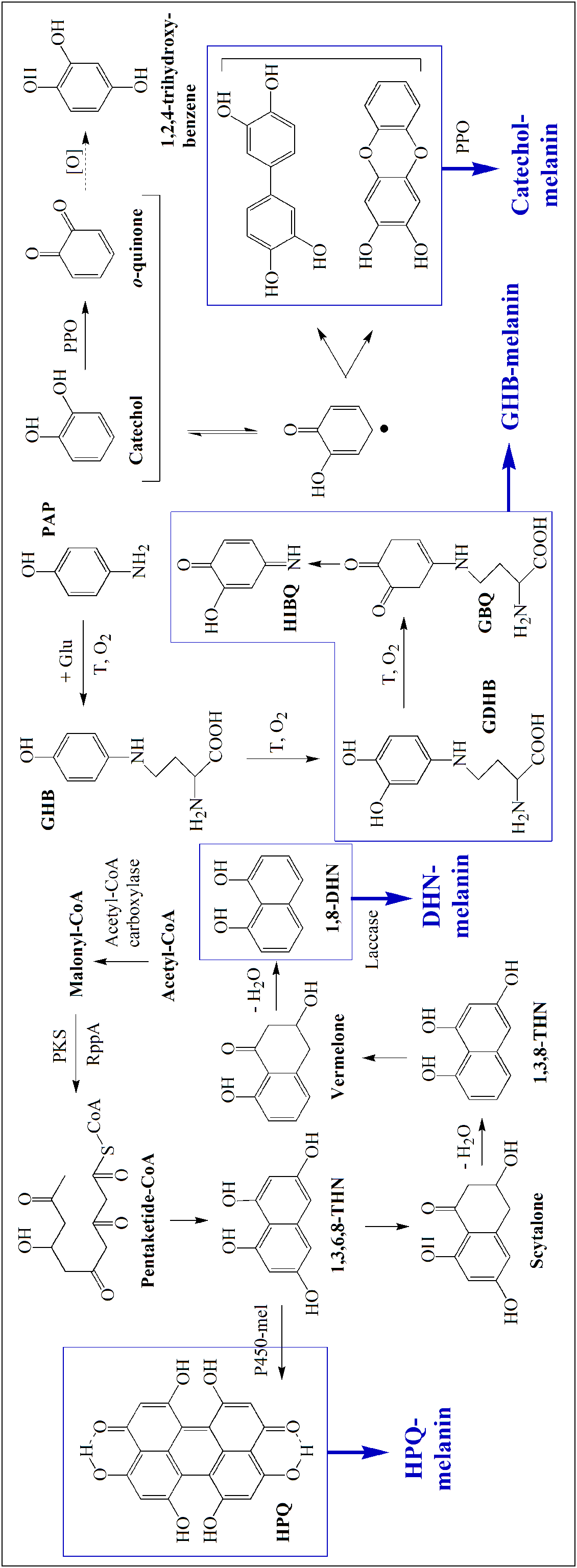
**

**Mechanism of hydroxylation** of 4-hydroxyphenylacetic acid (4-HPA) and formation of homogentisic acid (HGA) by a transposition of the acetic group (Hareland et al., 1975).

**
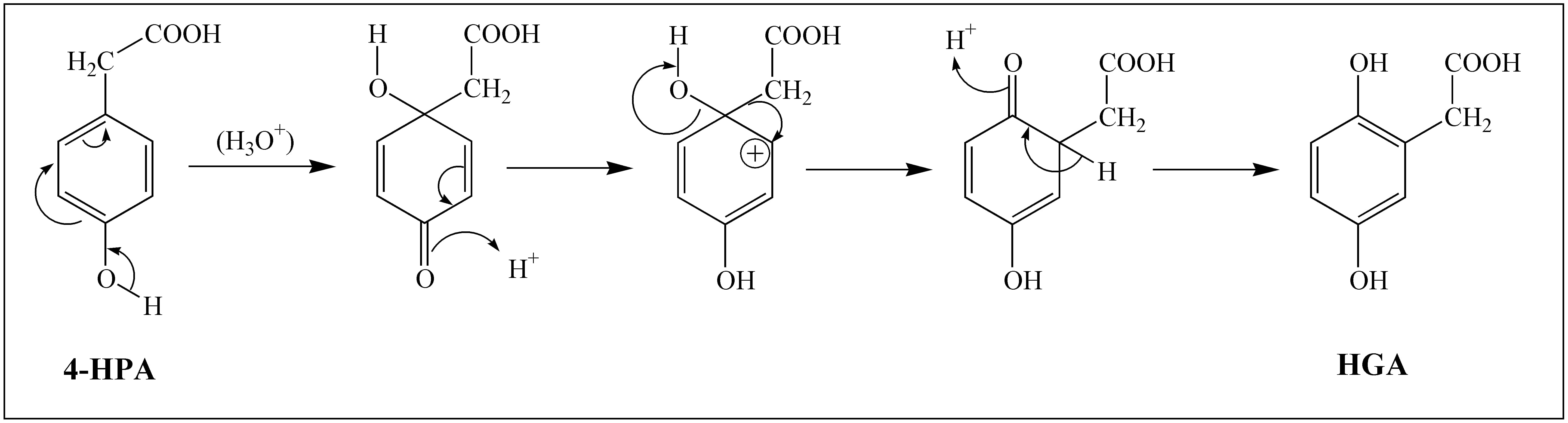
**


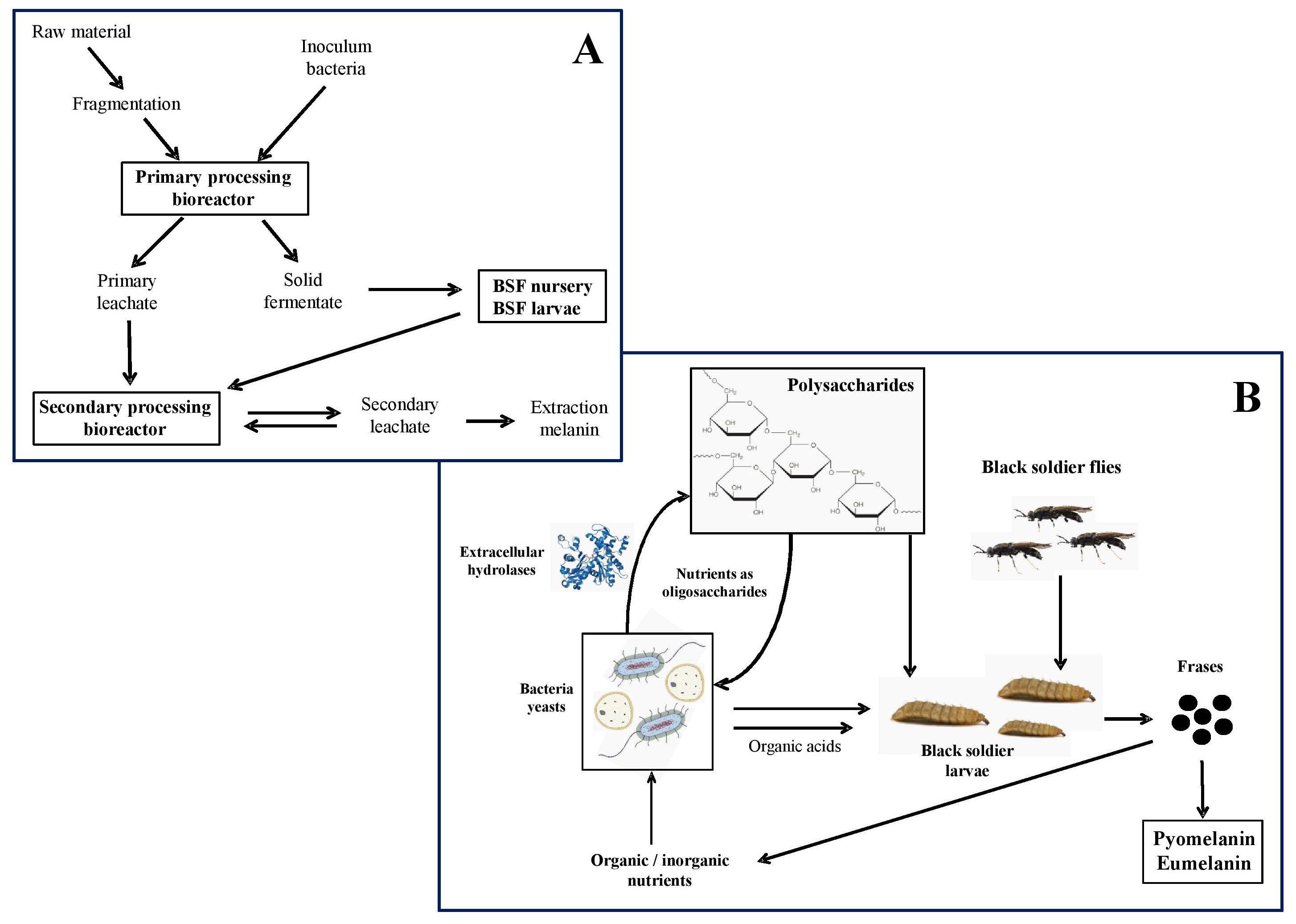


**The process for producing a pyomelanin-rich melanin mixture from organic waste-rich vegetal materials** (illustrated from the patent of Popa & Nealson, 2014; River Road Research, Inc., Tonawanda, USA). (**A**), general, (**B**), detailed procedure. Raw organic materials were first incubated in a primary bioreactor with microorganisms of the genera *Clostridium* or *Lactobacillus*. The primary leachate produced in the primary bioreactor was then converted into a secondary bioreactor into leachate rich in melanin by black soldier fly (BSF) larvae. During growth, the microorganism uptake nutrients and produce biomass and melanin, since BSF larvae grind partly digested polysaccharides which are partly converted into natural melanin or inorganic fertilizer, difficult to biodegrade and hence accumulate in the bioreactors. As a source of nutrients, leachates are provided from food or sugar-rich liquid wastes of the food industry and can be used raw or augmented with low-cost sugar-rich solutions such as molasses, hydrolyzed cellulose, or starch.


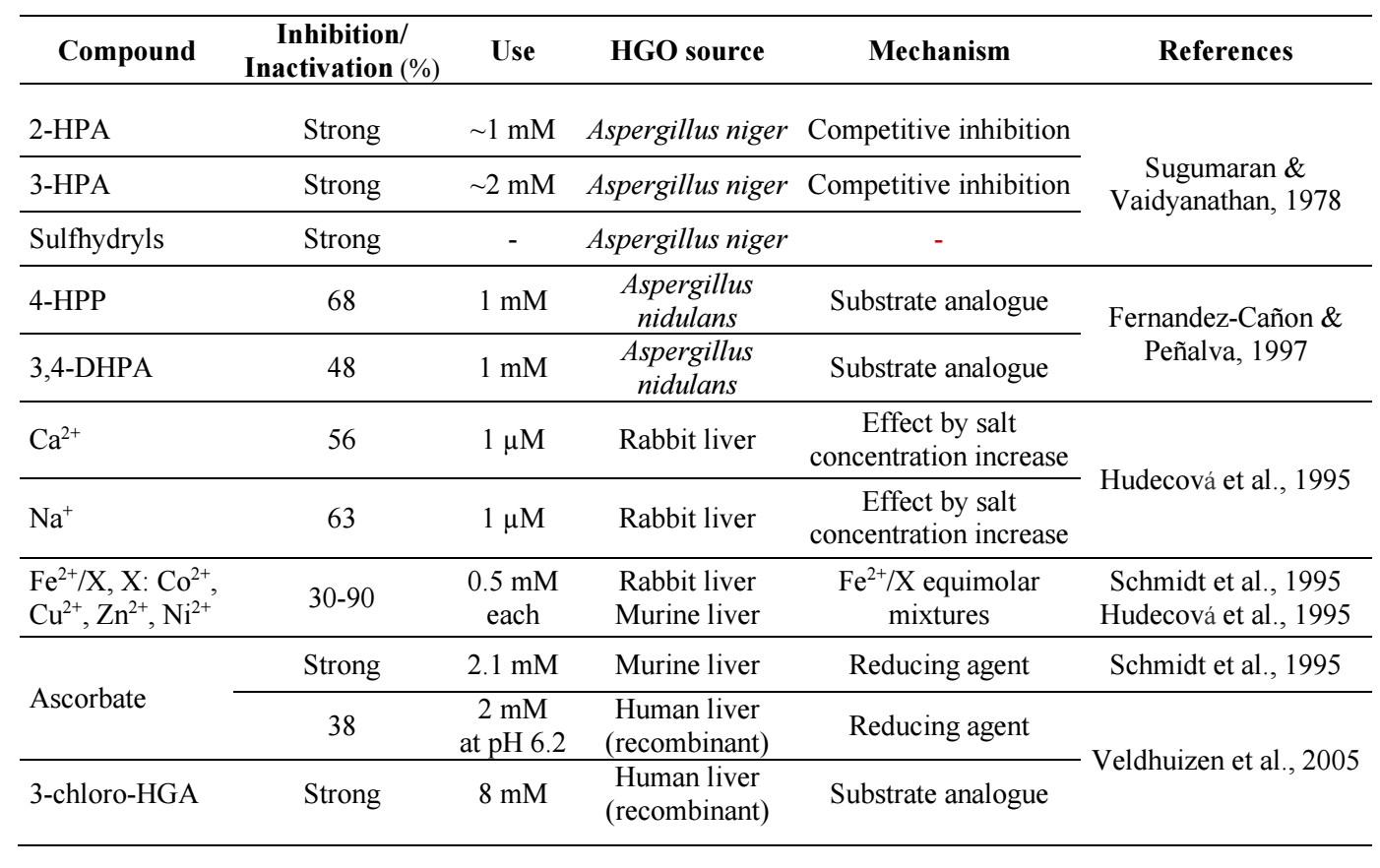


**Inhibitors and inactivating compounds of some purified HGA 1,2-dioxygenase (HGO)**. The selected compounds are those acting at the lowest concentrations. For this selection, Fe-chelating (-reacting) and toxic (Hg salts, cyanide) compounds had been discarded. Divalent ions or in combination with Fe^2+^ (such as Co, Zn, Cu, Ni) were successfully tested to inactivate purified mammal HGOs and might be used as well on microbial enzymes at appropriate concentrations. HPA, hydroxyphenylacetic acid; 4-HPP, 4-hydroxyphenylpyruvate; 3,4-DHPA, 3,4-dihydroxyphenylacetic acid. ‘-‘, not indicated.
